# Recent Progress of Organic–Inorganic Hybrid Perovskites in RRAM, Artificial Synapse, and Logic Operation

**DOI:** 10.1002/smsc.202100086

**Published:** 2021-11-10

**Authors:** Cheng Zhang, Yang Li, Chunlan Ma, Qichun Zhang

**Affiliations:** ^1^ Jiangsu Key Laboratory of Micro and Nano Heat Fluid Flow Technology and Energy Application School of Physical Science and Technology Suzhou University of Science and Technology Suzhou Jiangsu 215009 China; ^2^ Department of Materials Science and Engineering City University of Hong Kong Kowloon Hong Kong SAR 999077 China; ^3^ Center of Super-Diamond and Advanced Films (COSDAF) City University of Hongkong Hong Kong SAR 999077 China

**Keywords:** artificial synapses, logic operation, memristor, neuromorphic computing, organic-inorganic perovskite

## Abstract

Organic–inorganic hybrid perovskites (OHPs) with a rich reservoir of distinct physical attributes have been considered as promising resistive switching materials for next‐generation electronic devices, including resistive random‐access memory (RRAM) devices, artificial synapses, and logic operation. In this review, we first briefly introduce the structural and photoelectronic properties of OHPs materials, followed by elaborating the typical lateral/vertical device architectures and commonly‐used device fabrication technologies. Secondly, recent progress of OHPs in three categories of RRAM, artificial synapses, and logic operation, including various materials design, outstanding electrical behaviors, and multifunctional applications, are well discussed. Besides, the operational mechanisms and performance improvement strategies are summarized in a recapitulative way. Finally, current challenges and future development prospects of OHPs‐based devices are demonstrated to provide a better guideline for the next‐generation technical revolution. This review offers an encouraging recognition that OHPs hold immense expectation to keep up with the upcoming big‐data and artificial intelligence era.

## Graphical abstract

Organic–inorganic hybrid perovskites (OHPs) with numerous physical attributes have been considered as a promising contender for next‐generation information storage and processing technologies. Here, we offer a timely review about the recent progress of OHPs in RRAM, artificial synapses, and logic operation. The challenges and development prospects are also discussed.

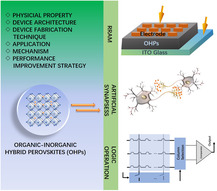

## 1 Introduction

In the era of booming the development of artificial intelligence (AI), the fifth generation networks (5 G), cloud services, and internet of things (IoT), a lot of high‐tech companies such as Hua Wei Technologies, Google Inc., and IBM, have offered the future visions of building an internet of everything. However, the traditional silicon‐based computing systems face a serious difficulty of scaling down to obtain faster operation rate and higher memory capacity, stemming from its physical and technical limits. Another huge hindrance to overcome is the von Neumann bottleneck, where the memory and central processing unit (CPU) are separately located in the conventional computers.^[^ ^1^ ^]^ Thus, novel computing primitives and data‐storage principles come to the front, with the traits of high energy efficiency, high‐frequency data transmission, massive information storage and faster processing physical speed. To address the aforementioned issues, some logic circuits such as resistive random‐access memory (RRAM) devices and memristor‐based artificial synapses have been proposed as the potential next‐generation devices for future electronic applications.^[^ ^2^ ^]^ Over the past decades, despite continuous research on metal‐oxides‐related devices, the shortcomings of energy‐extensive consumption, high‐temperature‐dependence fabrication, and poor flexibility, seem to be insurmountable.^[^ ^3^ ^]^ Thus, it is urgent to explore more advanced materials.

Halide perovskites (HPs) have emerged as great promising candidates for diverse optoelectronic applications, involving solar cells,^[^ ^4^ ^]^ photodetectors,^[^ ^5^ ^]^ luminescence‐based anti‐counterfeiting,^[^ ^6^ ^]^ light‐emitting diodes (LEDs),^[^ ^7^ ^]^ and field‐effect transistors (FETs).^[^ ^8^ ^]^ In contrast to some traditional organic and inorganic materials, HPs possess distinct properties of high charge mobility, long carrier‐diffusion length, simple fabrication process, magnetic and dielectric polarization, and so on.^[^ ^9^ ^]^ Besides, the solution‐processable and fast crystallization nature are conducive to produce denser and more uniform perovskite films. Significantly, the ubiquitous hysteresis eﬀects in HPs have been regarded as an intriguing advantage for RRAM, artificial synaptic emulation and neuromorphic computing. Till now, several types of HPs have been developed for practical electronic applications, which have been customarily classified into organic‐inorganic hybrid perovskites (OHPs, e.g., FA_0.83_MA_0.17_Pb(I_0.82_Br_0.18_)_3_, and MA_3_Bi_6_I_9_) or inorganic halide perovskites (IHPs, e.g., CsPbI_3_, CsSnI_3_, CsBi_2_I_9_, and NaSbS_2_), 2D (2D) halide perovskites (Ruddlesden‐Popper perovskites, e.g., (MTEA)_2_(MA)_4_Pb_5_I_16_) or 3D (3D) halide perovskites (e.g., MAPbI_3_), lead‐based perovskites (e.g., MAPbBr_3_) or lead‐free perovskites (e.g., CsSnI_3_), according to different division principles (Notes: MA = CH_3_NH_3_^+^, methylammonium; FA = HC(NH_2_)_2_^+^, formamidinium).^[^ ^10^ ^]^

Compared to IHPs, OHPs exhibit more noticeable advantages of tunable bandgap, satisfactory solution processability, high mechanical flexibility, and excellent ions/defects migration properties, due to the presence of organic ammonium cation groups.^[^ ^11^ ^]^ Attributed to the dipolar cation interaction, high‐crystallinity OHP films or polarized OHP domains could be easily obtained. So far, various OHPs have been spotlighted for RRAM and artificial synapses, involving commonly‐used 3D MAPbI_3_, MAPbCl_3_, MAPbBr_3_, and some 2D Ruddlesden‐Popper perovskites. Recently, OHPs endow the traditional optoelectronic devices a new life with fast switching response (~100 ns), high‐density data storage (>0.1 TB in.^−2^), low energy consumption (<100 fJ μm^−2^), simple device‐preparation techniques, low operational voltages (<1 V), long retention time (>10^4 ^s), and high ON/OFF ratio (>10^3^).^[^ ^12^ ^]^ In general, OHPs materials of excellent ferroelectric, dielectric, light‐sensitive and semiconducting properties are merging as great contenders for RRAM and artificial synapse applications.

**Figure 1:**
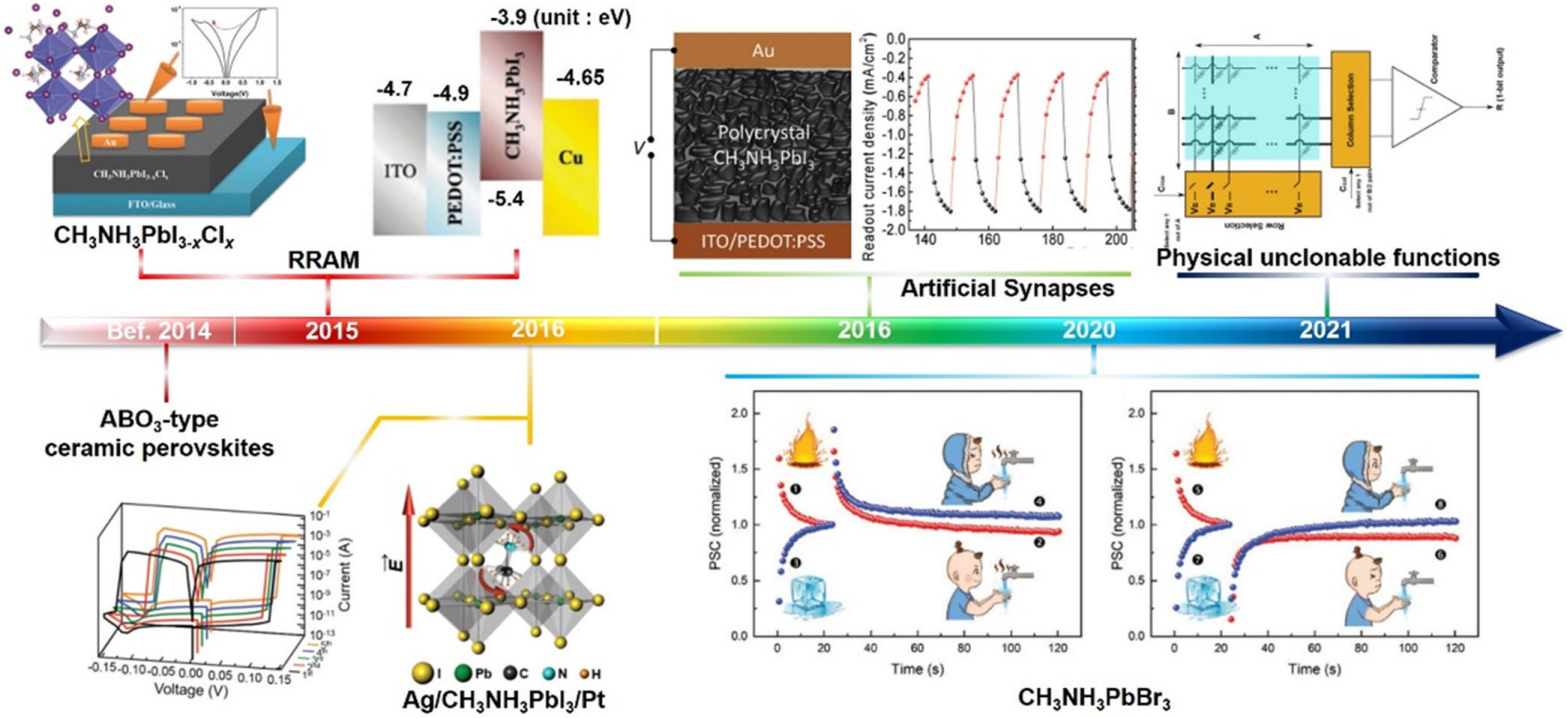
A brief timeline of the development of OHPs‐based RRAM, artificial synapses and logic operation. “CH_3_NH_3_PbI_3_‐xClx” Reproduced with permission.^[^ ^13^ ^]^ Copyright 2015, Wiley‐VCH. “Ag/CH_3_NH_3_PbI_3_/Pt” Reproduced with permission.^[^ ^14^ ^]^ Copyright 2016, Wiley‐VCH. “Artificial Synapses” Reproduced with permission.^[^ ^15^ ^]^ Copyright 2016, Wiley‐VCH. “CH_3_NH_3_PbBr_3_” Reproduced with permission.^[^ ^16^ ^]^ Copyright 2020, Wiley‐VCH. “Physical unclonable functions” Reproduced under the terms of the CC‐BY 4.0 license.^[^ ^17^ ^]^ Copyright 2021, The Authors, published by Springer Nature.

In this review, we mainly focus on the recent progress of OHPs in RRAM, artificial synapses, and logic operation. **Figure** **1** chronologically depicts a brief development schedule of the electronic devices.^[^ ^13^, ^14^, ^15^, ^16^, ^17^ ^]^ The intrinsic physical properties of OHPs are first introduced to understand their structural and optoelectronic characteristics. In the subsequent sections, three typical device architectures and commonly‐used devices fabrication technologies are described. For the detailed OHPs‐based applications, RRAM devices (grouped into bistable or multilevel resistive switching, multifunctional applications (i.e., capacitance effect, humidity effect, light‐response effect and flexibility)), artificial synapses, neuromorphic computing, and logic operation, are well discussed. Besides, the memory mechanism and performance improvement strategies are summarized in a recapitulative way. Finally, we provide current challenges and development prospects of OHPs‐based devices and anticipate a bright future to transcend silicon‐based semiconductors.

## 2 Structural and Photoelectronic Properties of OHPs

**Figure 2:**
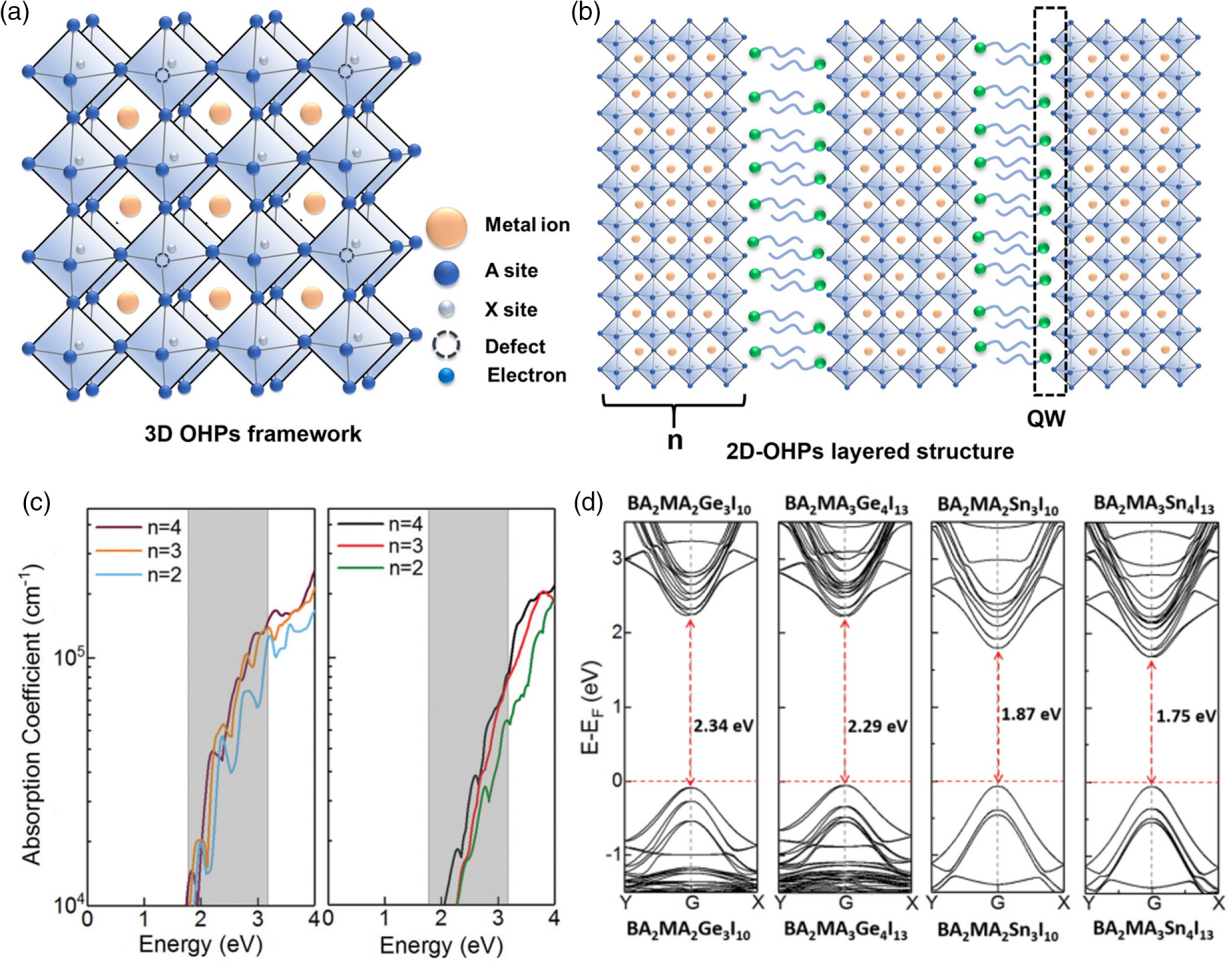
a) Chematic structures of 3D OHPs framework. b) Schematic structures of 2D layered OHP structure. c) The computed light absorption spectra of BA_2_MA_*n*−1_Sn_n_I3_*n*+1_ (left) and BA_2_MA_*n*−1_Ge_n_I_3*n*+1_ (right). d) The computed band structures of BA_2_MA_*n*−1_M_*n*_I3_*n*+1_ (*M* = Ge or Sn, *n* = 3–4) based on the PBE0 + SOC scheme. Reproduced with permission.^[11b]^ Copyright 2018, Royal Society of Chemistry.

The tunable composition is one of the extraordinary properties of OHPs, leading to a wide variety of material categories with the chemical formula of ABX_3_, A_3_B(III)_2_X_9_, A_2_ B(I)B(III)_2_X_6_, (A*)_2_A_*n*−1_B_*n*_X_3*n*+1_ and so on. In the ABX_3_ formula, OHPs commonly present a 3D octahedral framework, where metal halide is posited at the lattice corner (**Figure** **2**a), and A, B, X sites can be replaced by different elements. When A site is substituted by monovalent organic alkylammonium cations (e.g., MA^+^, FA^+^, n‐butylammonium (n‐BA^+^), and 2‐phenylethylammonium (PEA^+^)), the bond length between B and C sites can be modified accordingly, as well as the exotic bandgaps. For the 2D organic layered perovskite (2D OHPs) with a general chemical formula of (A*)_2_A_*n*–1_B_*n*_X_3*n*+1_, A* and A are the long‐chain organic cation (spacer, e.g., n‐BA^+^ and PEA^+^) and short‐chain organic cation (e.g., MA^+^ and FA^+^), respectively. 2D OHPs show a sandwiched structure with two long organic spacers and n layers of inorganic [BX_6_] octahedral sheets (Figure 2b). Considering that the barriers of organic ligands and inorganic perovskite layers present high contrast in dielectric constants, the natural quantum well (QW) was proposed to describe this mismatch in 2D OHPs. As an intrinsic property of 2D OHPs, QW can screen Coulomb interaction due to the dielectric confinement effect, leading to a strong electron‐hole interaction with large oscillator strength, high exciton binding energy, and 2D quantum confinement effect.^[4b]^ However, it should also be noted that the unique layered architecture of 2D OHPs may suffer from a mixture of multiple quantum‐wells (MQW) structures with different well widths, which could limit their further applications in the photoelectric field.^[4b]^ As previously reported, the nanostructure of OHPs could be transformed from 2D layer ((A*)_2_A_*n*–1_B_n_X_3*n*+1_) to 3D framework (ABX_3_) by introducing long alkylammonium cations at the A site. For instance, the 2D layer structure of (CH_3_(CH_2_)_3_NH_3_)_2_(CH_3_NH_3_)_*n*−1_Pb_*n*_I_3*n*+1_ could be constructed through regulating n from 1 to 4.[^4^, ^18^] Thus, designing a bi‐cation (e.g., (RNH_3_)_2_(MA)_*n*−1_Pb_*n*_I_3*n*+1_ and [CH_3_(CH_2_)_8_‐NH_3_]_2_(MA)_*n*−1_Pb_*n*_Br_3*n*+1_) or triple‐cation perovskites (e.g., Cs_0.05_FA_0.79_MA_0.16_Pb(I_*n*_Br_1−*n*_)_3_) becomes one possible strategy for tuning the photoelectronic properties.^[^ ^19^ ^]^ The B sites can also be filled by smaller divalent metallic elements (e.g., toxic Pb^2+^, low‐toxic Sn^2+^ and Cu^2+^), while C sites can be substituted by halide ions (Cl^−^, Br^−^, and I^−^) or non‐halogen ions (BF_4_^−^, PF_6_^−^, and SCN^−^). Thus, it can be learned that OHPs own attractive advantages of tunable components (e.g., metal ions, polar organic cations, and counterions), outstanding ionic nature, high symmetry, high electronic dimensionality, and strong spin‐orbit coupling property.

Attributed to the above‐mentioned structural characteristics, OHPs exhibit superior photoelectronic properties, including low exciton binding energies, high optical absorption coefficients, coordinated electron and hole mobilities, and long photogenerated carrier lifetimes, which are desirable for the highly‐efficient photoelectronic devices. For instance, Zeng et al. implemented a theoretical assessment on the Sn‐ and Ge‐based 2D Ruddlesden‐Popper OHPs of BA_2_MA_*n*−1_M_*n*_I_3*n*+1_ (M = Sn or Ge, *n* = 2–4).^[11b]^ It was found that the exciton binding energies and bandgaps decreased as the layer thickness (*n*) of the 2D perovskites increased from 2 to 4. Figure 2c showed that the light absorption of Ge‐ and Sn‐based 2D OHPs over the visible light range (wavelengths from 390 to 700 nm) was enhanced with the increased n layer. Notably, the light absorption of Sn‐based 2D OHPs possessed smaller fundamental bandgaps than that of Ge‐based OHPs. Due to the existence of the protective organic ligand layer, 2D OHPs have superior temporal stability to their 3D analogs. The calculated electronic band structures of BA_2_MA_*n*−1_M_*n*_I_3*n*+1_ (*n* = 3–4) are depicted in Figure 2d, demonstrating that all of the 2D BA_2_MA_*n*−1_M_*n*_I_3*n*+1_ OHPs are presented as direct‐gap semiconductors. These features of Sn‐ and Ge‐based 2D OHPs are expected to fabricate high‐performance photovoltaic and/or photoelectronic applications.

## 3 Typical Device Architectures

**Figure 3:**
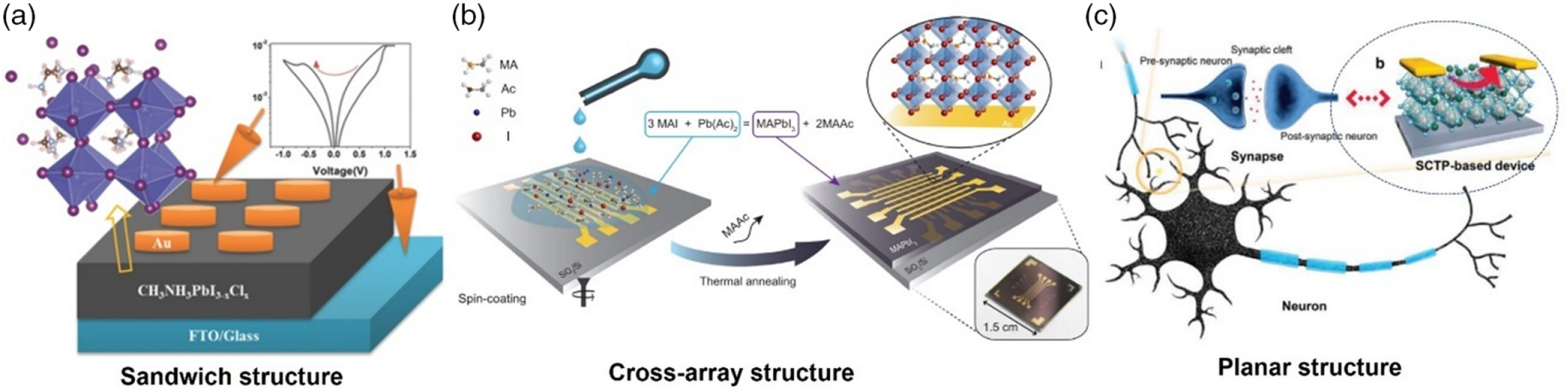
Three typical device architectures of “Sandwich”, “Cross‐array”, and “Planar” structures. “Sandwich structure” Reproduced with permission.^[^ ^13^ ^]^ Copyright 2015, Wiley‐VCH. “Cross‐array structure” Reproduced with permission.^[^ ^21^ ^]^ Copyright 2019, Wiley‐VCH. “Planar structure” Reproduced with permission.^[^ ^16^ ^]^ Copyright 2020, Wiley‐VCH.

Three typical device architectures, including top‐bottom sandwich structure, left‐right planar structure, and cross‐array structure, are generally adopted to prepare these electronic devices, as illustrated in **Figure** **3**. Specifically, the top‐bottom “sandwich” and “cross‐array” structures can be classified into vertical metal–insulator–metal (MIM) category, which can provide massive device cell arrays and high‐density data storage. The sandwich configuration has been widely investigated for basic research in laboratory (Figure 3a),^[^ ^13^ ^]^ due to its unique advantages of easy preparation, low cost, and so on.^[^ ^20^ ^]^ For the cross‐bar array geometry with the structure of parallel bottom electrode lines (word lines) and orthogonal top electrode lines (bit lines), it possesses more potential to scale down every single device cell and realize a highly integrated RRAM architecture. Figure 3b shows an example of a cross‐bar array RRAM device, using MAPbI_3_ as the active layer and Au as the top and bottom electrodes. The OHPs‐based device array of 8 × 8 was integrated into a cross‐bar array architecture, achieving 94% yield and stable memory cell characteristics.^[^ ^21^ ^]^ In addition, when external diodes are connected to the neighboring cells, the selective operation of memory cells can be well realized by suppressing the cross‐talk interference.

Unlike the MIM structural device, the planar structure has two transversely distributed electrodes and an OHPs‐formed channel, which is analogous to the lateral field‐effect transistor structure. Thus, the planar configuration holds the architectural advantage of being compatible with commercial complementary metal‐oxide‐semiconductor (CMOS) circuits. For example, as displayed in Figure 3c, Xu et al. demonstrated a high‐quality MAPbBr_3_‐based single‐crystalline thin platelets synaptic device with a planar structure.^[^ ^16^ ^]^ Based on this device, the essential synaptic behaviors of short‐term potentiation/depression (STP/STD), paired‐pulse facilitation (PPF) and spike‐time‐dependent plasticity (STDP), can be simulated for the application of neuromorphic electronics.

## 4 Device Fabrication Techniques

After years of unremitting efforts, OHP films have been successfully fabricated by various techniques, including wet methods (such as spin‐coating, dip‐coating, casting, electrochemical deposition, ink‐jet printing, spray pyrolysis, and interdiffusion growth) and dry methods (e.g., atomic layer deposition, thermal evaporation method, and vapor deposition).^[^ ^22^ ^]^ Among these methods, spin‐coating and vapor deposition methods are the most frequently used methods, attributed to their merits of high efficiency and suitability for large‐area facilitation.^[10d]^ Recently, more advanced techniques have been developed to meet several special requirements. For example, Wakamiya et al. combined hot antisolvent treatment (HAT) and solvent vapor annealing (SVA) methods to increase nucleation sites of OHPs.^[^ ^23^ ^]^ Jiang et al. introduced a cation exchange method of converting 3D MASnI_3_ to 1D HASnI_3_.^[^ ^24^ ^]^ These techniques significantly diversify the application scenarios of OHP films.

### 4.1 Spin Coating Method

**Figure 4:**
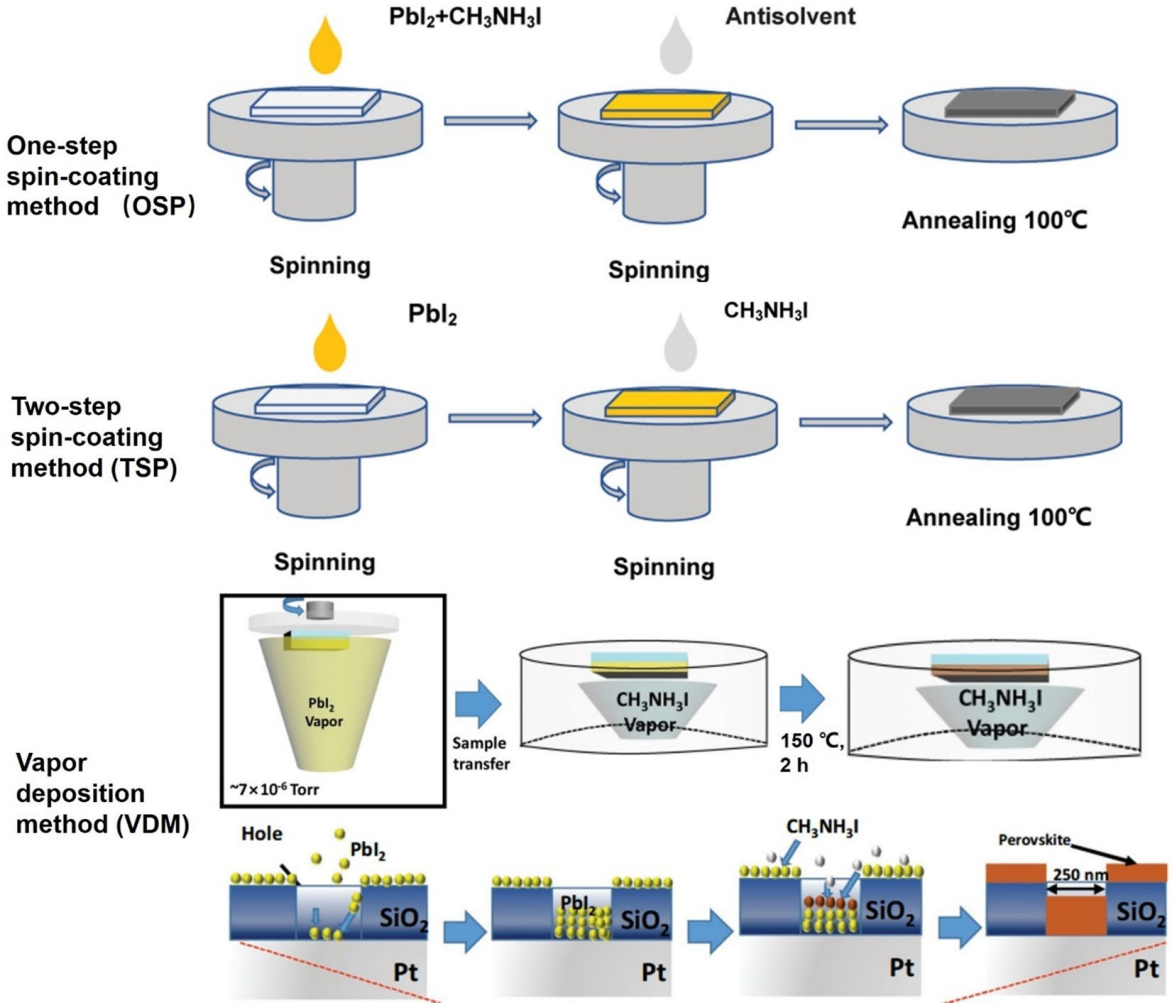
Commonly‐used OHPs‐based film fabrication techniques: One‐step‐coating method (OSP, top) and two‐step spin‐coating method (TSP, middle); Vapor deposition method (VDM, bottom). Reproduced with permission.^[^ ^25^ ^]^ Copyright 2017, Wiley‐VCH.

The spin‐coating method (SPM) is widely used in the fabrication of OHP films, due to its simple operation, controllable film quality and thickness, and low temperature. More specifically, the SPM method can be divided into two types: one‐step spin‐coating method (OSP) and two‐step spin‐coating method (TSP). For the OSP method (**Figure** **4**, top), OHP materials are prepared by a solution‐processable method in advance. Ionic liquid (e.g., methylamine formate and methylamine acetate), *N,N*‐dimethylformamide (DMF) and dimethyl sulfoxide (DMSO), are commonly employed as organic solvents to dissolve metal halide and OHPs. Accompanied by the assistance of antisolvents (e.g., toluene, chlorobenzene, and diethyl ether) and annealing post‐treatment, OHPs film could be rapidly fabricated in hours. The film thickness and morphology highly rely on solvent volatilization speed, solution viscosity, spin‐coating rate, and interfacial interaction force. For the TSP method (Figure 4, middle), the metal halide in DMF or DMSO and organic amine salts in isopropyl alcohol (IPA) are spin‐coated onto the substrate in sequence, where a further reaction will proceed through the direct contact between the two phases. Given that tin iodide is soluble in IPA, the TSP method is incapable of fabricating Sn‐based OHP films.

### 4.2 Vapor Deposition Method

Vapor deposition methods (VDM), involving hybrid chemical deposition, thermal evaporation deposition, dual‐source vacuum deposition and sequential vapor deposition, have also been adopted to deposit high‐quality and scalable OHP films. Compared to the solution processing method, although VDM can precisely control the thickness of OHP films through monitoring the deposition rate, it requires a vacuum condition and no contamination to the vacuum chamber. Lee et al. produced a 16 × 16 cross‐bar array RRAM device by sequential VDM, in which MAPbI_3_ was deposited into hole structures on silicon wafers substrate (Figure 4, bottom).^[^ ^25^ ^]^ After the deposition of the first layer PbI_2_, the substrate was placed into a sealed petri dish followed by the sublimation of CH_3_NH_3_I powder under 150 °C. The as‐fabricated MAPbI_3_‐based RRAM device exhibited excellent characteristics of low operation voltage (1/−1 V), fast switching speed (200 ns), 500 endurance cycles, and long data retention (>10^5^ s). Wu et al. also utilized vapor conversion synthesis to fabricate a MAPbBr_3_‐based RRAM device and subsequently confirmed that a chemical gradient of MAPbBr_3_ was generated due to the insufficient MABr diffusion, resulting in an unusual gradient Fermi level distribution and a Schottky contact to form a current rectification.^[^ ^26^ ^]^

## 5 OHPs‐Based Memory Applications

### 5.1 Resistive Random‐Access Memory (RRAM)

RRAM devices are constructed based on the resistive switching (RS) effect triggered by the electric field, illumination, magnetic field, and temperature conditions. Over the past decades, advanced organic and inorganic materials have been developed and applied to study the RS effects.^[^ ^27^ ^]^ Some typical characteristics of bipolar switching properties, multilevel tunability of resistance states and other external‐stimuli induced behaviors, were reported in a number of investigations.^[^ ^28^ ^]^ To establish an evaluation criteria for the RRAM performance (stability, reproducibility, energy conservation, ect.), some physical parameters are considered, including current ratio (10–10^9^) between a high‐resistance/low‐conductivity state (HRS/OFF state) and a low‐resistance/high‐conductivity state (LRS/ON state), operational voltage (as low as possible), switching speed (10–100 ns), endurance cycles (10^3^–10^7^), and retention ability (10^3^ s–10 years).^[^ ^29^ ^]^ Owning to the co‐existence and coupling of electronic and ionic constituents, OHPs gather numerous virtues of inherently localized doping, tunable carrier concentrations, and superior dielectric constants, which are extremely suitable for next‐generation RRAM devices.

#### 5.1.1 Bistable Resistance State Switching

**Figure 5:**
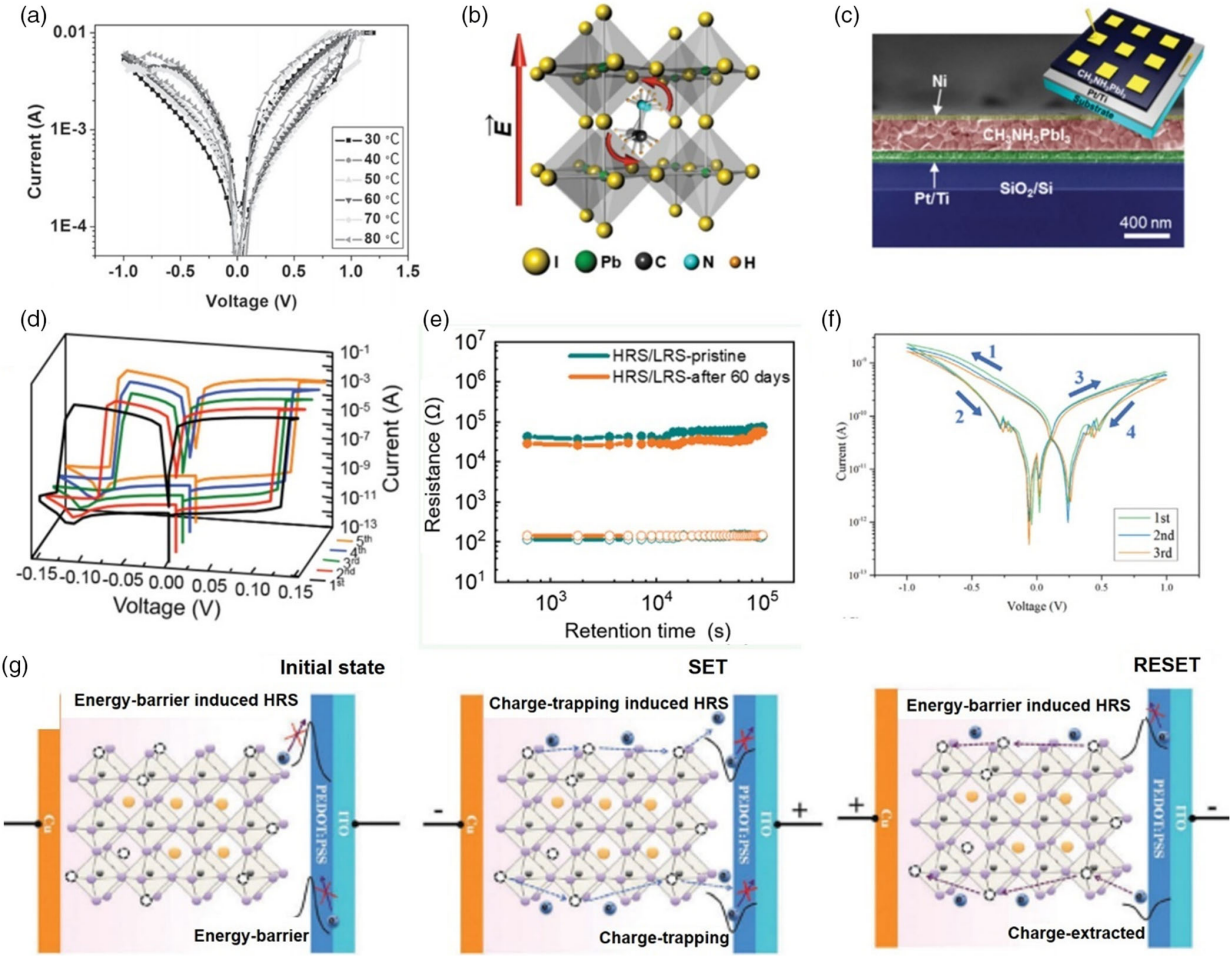
a) Temperature‐dependent *I–V* curves of the Au/MAPbI_3−*x*_Cl_*x*_/FTO device. Reproduced with permission.^[^ ^13^ ^]^ Copyright 2015, Wiley‐VCH. b) The perovskite crystal structure of MAPbI_3_. c) Cross‐sectional SEM image of the MAPbI_3_‐based device. d) *I–V* characteristics of Ag/MAPbI_3_/Pt cells. Reproduced with permission.^[^ ^14^ ^]^ Copyright 2016, Wiley‐VCH. e) Retention characteristics of the pristine device and after‐60‐days device. Reproduced with permission.^[19a]^ Copyright 2020, American Chemical Society. f) *I–V* curves of Al/CsFAMAPbIBr/ITO device. Reproduced with permission.^[19b]^ Copyright 2020, Wiley‐VCH. g) The process of electron flowing and barrier switching under the external bias. Reproduced with permission.^[^ ^31^ ^]^ Copyright 2015, Royal Society of Chemistry.

Although ABO_3_‐type ceramic perovskite structures were reported decades ago, the OHPs‐based RRAM remained unexplored until 2014. Huang et al. firstly observed the RRAM properties with an ON/OFF current ratio of 10^4^, paving the way for solar cells design and memristors/circuits exploitation.^[^ ^30^ ^]^ Subsequently, Wang et al. fabricated the first case of OHP‐based (MAPbI_3−*x*_Cl_*x*_) nonvolatile memory devices (**Figure** **5**a).^[^ ^13^ ^]^ Bipolar RS behaviors were demonstrated in Au/MAPbI_3−*x*_Cl_*x*_/FTO structured devices with the low set/reset voltage of 0.8/−0.6 V, long retention time (>10^4^ s) and high endurance cycles (>100 times), which are comparable to typical oxide‐based devices. More importantly, the MAPbI_3−*x*_Cl_*x*_ based devices showed good thermal stability and high reproducibility, even under the condition of different testing positions, temperatures (30–80 °C) and film thicknesses (2.5, 2, and 1 μm).

Since then, OHPs‐based RRAM has caused numerous research attention. Especially, MAPbI_3_, as one of the outstanding OHP representatives, has been extensively studied due to the definite lattice parameters and easy processing. Unlike the Pb^2+^ cation and I^–^ anion in spherical shapes, the MA^+^ cation shows asymmetric dumbbell shapes and slants toward the diagonal direction by 30° in a crystal cell (Figure 5b). Thus, the MA^+^ cation can rotate at the condition of an external electric field along the c‐axis. In 2015, Sun et al. discovered the photoconductive and memristive effect in the device of indium tin oxide (ITO)/poly(3,4‐ethylenedioxythiophene)polystyrene sulfonate (PEDOT:PSS)/MAPbI_3_/metal (Cu, Au or ITO), indicating a potential application in integrated logic circuits.^[^ ^31^ ^]^ As summarized in **Table** **1**, the ITO/PEDOT:PSS/MAPbI_3_/Cu device exhibited a well repeatable RRAM performance with the ON/OFF ratio 10^4^ at a read‐out voltage of 50 mV.^[^ ^32^, ^33^, ^34^, ^35^, ^36^, ^37^, ^38^ ^]^ However, Cu/MAPbI_3_/Cu and Cu/MAPbI_3_/Au devices showed no memory behavior, elucidating that the RRAM phenomenon was originated from the barrier on the PEDOT:PSS/MAPbI_3_ interface. To explain the RRAM behavior, a mechanism of the barrier modification induced by charge trapping, was proposed and illustrated in Figure 5g. When a negative voltage was applied to the device, the charges were extracted from Cu cathode to MAPbI_3_ to break the charge equilibrium. Hence, the barrier of PEDOT:PSS/MAPbI_3_ interface was vanished, leading to the formation of conducting path and HRS‐to‐LRS state transition (Set process). When a positive voltage was applied, the charges were extracted from MAPbI_3_ to restructure the barrier (Reset process). As depicted in Figure 5c,d, the performance of MAPbI_3_‐based device was greatly optimized by Jang et al. with the fabrication of Ag/MAPbI_3_/Pt, showing low set/reset voltages (0.13/−0.13 V) and high ON/OFF ratios (10^6^) induced by ±0.15 V pulses.^[^ ^14^ ^]^

**Table 1:** Summary of the performance in MAPbI_3_‐based RRAM devices

Device structure	Perovskite	Processing technique	Set/reset voltage	Current ratio	Retention [s]	Endurance [times]	References
Cu/OHPs/PEDOT:PSS/ITO	MAPbI_3_	TSP; 100 °C, 2 h	−1.0/2.0	10^3^	3 × 10^4^	3000	[31]
Note: Cu/MAPbI_3_/Cu and Cu/MAPbI_3_/Au show no RRAM phenomenon; ITO/PEDOT:PSS/ MAPbI_3_/ITO, ITO/PEDOT:PSS/CH_3_NH_3_PbI_3‐x_Cl_x_/Cu and ITO/PEDOT:PSS/CH_3_NH_3_Pb_0.5_Sn_0.5_I_3_/Cu show RRAM phenomenon; ITO/PEDOT:PSS/ MAPbI_3_/Au shows unipolar RRAM phenomenon							[31]
Ag/OHPs/Pt	MAPbI_3_	OSP; 70 °C, 30 min	0.13/−0.13	10^6^	1.1 × 10^4^	350	[14]
Au/OHPs/ITO/PET	MAPbI_3_	OSP; 110 °C, 15 min	0.7/−0.61	10	10^4^	400	[32]
Au/OHPs/Pt	MAPbI_3_	VDM; 110 °C, 2 h	1.0/−1.0	10^4^	10^5^	500	[25]
Ag/OHPs/Pt	MAPbI_3_	OSP; 100 °C, 10 min	0.18/−0.1	10^6^	3 × 10^4^	10^3^	[33]
Au/OHPs/ITO	MAPbI_3_	OSP;100 °C, 5 min	0.7/−0.61	10^1.5^	10^4^	600	[34]
Au/OHPs/ITO	MAPbI_3_	Thermal evaporation; 120 °C, 1 h.	−0.5/0.5	10^3^	10^4^	1000	[26]
Ag/OHPs/FTO	MAPbI_3_	OSP; 100 °C, 10 min	1.0/−1.0	10^6^	10^5^	1000	[35]
Au/OHPs /Graphene	MAPbI_3_	OSP; 100 °C, 5 min	0.68/−0.5	10^1.5^	10^4^	500	[36]
Al/PMMA/OHPs/PEDOT:PSS/ITO	MAPbI_3_	TSP; 100 °C, 10 min	0.5/−1.2	10^3^	10^4^	170	[37]
Au/OHPs/Au	MAPbI_3_	OSP; 100 °C, 5 min	0.96/0.55	10^8^	10^4^	1000	[21]
Au/OHPs /FTO/PET	MAPbI_3_	TSP; 100 °C, 30 min	0.5/−0.5	10	–	500	[38]

Nevertheless, MAPbI_3_ with low structural phase transition (55 °C) shows high sensitivity to moisture and temperature, which may lead to a degradation reaction from MAPbI_3_ to HI, CH_3_NH_2_ and PbI_2_. Several improvements have been designed to enhance the thermal and structural stability of OHPs. One of the solutions is to develop triple or multiple cation perovskite materials, which has been confirmed as a novel compositional strategy in the field of OHPs‐based solar cells by Grätzel and coworkers.^[^ ^39^ ^]^ Recently, Huang et al. attempted to apply triple‐cation OHPs (Cs_0.05_FA_0.79_MA_0.16_Pb(I_*n*_Br_1−*n*_)_3_) into RRAM application based on Au/OHP/ITO device, affording endurance cycles as high as 10^3^ times and retention time of 10^5^ s at the compliance current (*I*_cc_) of 100 mA (Figure 5e).^[19a]^ According to the kinetic Monte Carlo simulation and experimental support, they proposed a filament‐dominated RS mechanism, where Au electrodes acted as an iodine reservoir to suppress the diffusion of iodine ions, and hence formed iodine‐vacancy‐induced conductive filaments (CFs). In 2021, Wang et al. reported a similar multiple cation perovskite of Cs_0.05_(FA_x_MA_1−*x*_)_0.95_PbI_y_Br_3−*y*_ (CsFAMAPbIBr).^[19b]^ As shown in Figure 5f, the as‐fabricated Al/CsFAMAPbIBr/TiO_2_/FTO exhibited excellent hysteresis loops, demonstrating a great promise to further implement complex synaptic plasticity.

#### 5.1.2 Multilevel Resistance State Switching

**Figure 6:**
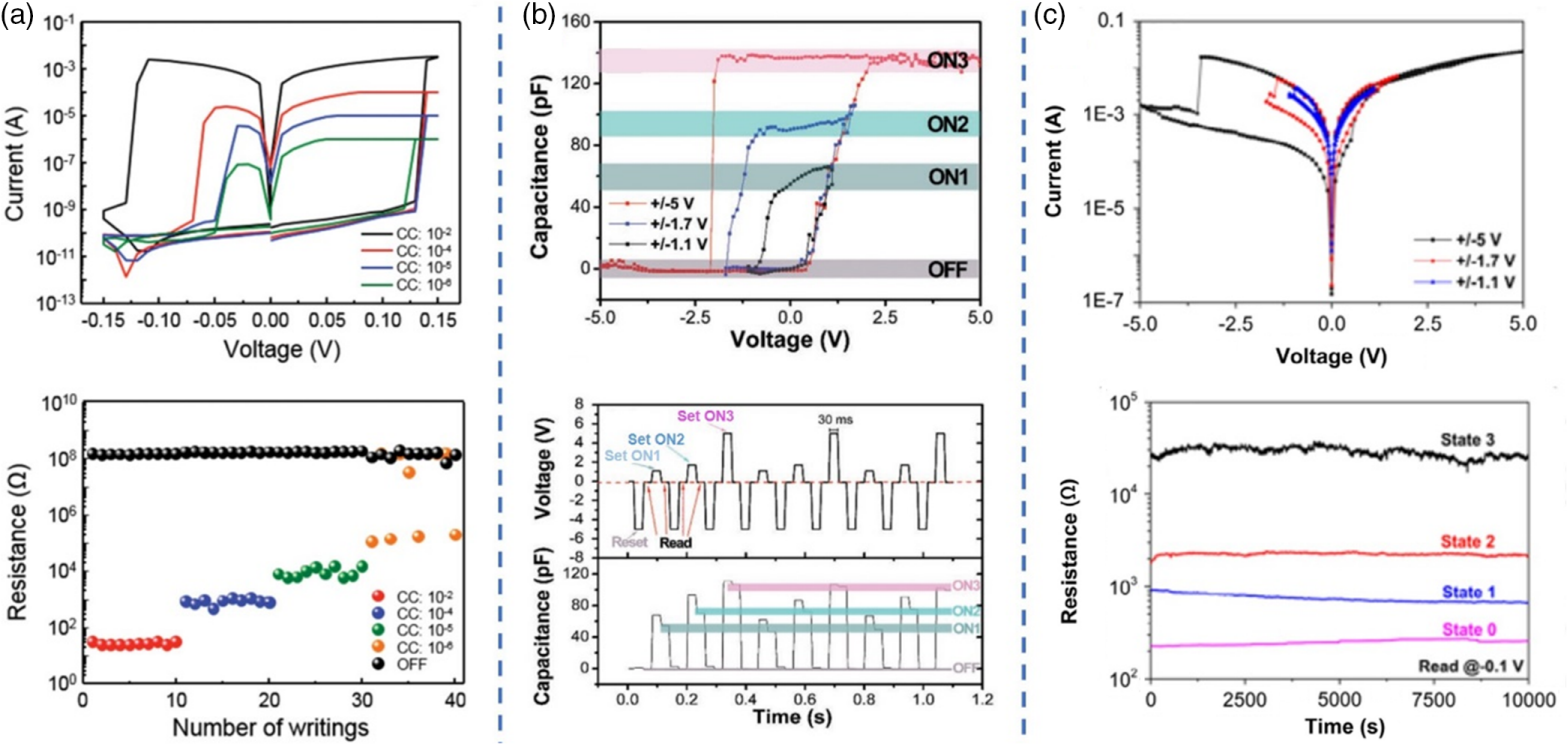
a) Multilevel resistive switching properties of Ag/MAPbI_3_/Pt cells under four different *I*_cc_. Reproduced with permission.^[^ ^14^ ^]^ Copyright 2016, Wiley‐VCH. b) Typical quaternary capacitance‐voltage loops of ITO/MASnBr_3_/Au device. Reproduced with permission.^[^ ^42^ ^]^ Copyright 2019, Wiley‐VCH. c) Multilevel operation of the MASnBr_3_‐based memristor under different *I*_cc_ and applied voltages. Reproduced under the terms of the CC‐BY 4.0 license.^[^ ^43^ ^]^ Copyright 2019, The Authors, published by John Wiley & Sons Australia, Ltd on behalf of UESTC.

Driven by traditional electronic device miniaturized limits, developing multilevel resistance state switching has been considered as a prospective strategy to exponentially increase the information storage density per unit area. By far, ternary (“0”, “1”, “2”), quaternary (“0”, “1”, “2”, “3”), and even higher multilevel memory behaviors have been achieved in oxides‐ or organic D‐A‐type materials‐based RRAM devices, which may significantly improve the amount of data storage.^[^ ^40^ ^]^ Although a diversity of OHPs has been reported to demonstrate RRAM behaviors, only several examples were utilized for multilevel memory applications. Jang et al. provided a notable paradigm of quaternary resistive switching behavior from Ag/MAPbI_3_/Pt device cells under four different *I*_cc_ of 10^−2^, 10^−4^, 10^−5^, and 10^−6^ A, which was in accordance with the pulse‐induced reversible resistive switching results (**Figure** **6**a).^[^ ^14^ ^]^ Analogously, Lee et al. achieved multilevel data storage with four resistance states based on a lead‐free bismuth (Bi) OHP material of MA_3_Bi_2_I_9_. The Au/MA_3_Bi_2_I_9_/ITO device displayed fast switching speed (100 ns), long retention time (10^4^ s) and reliable endurance properties (300 cycles).^[^ ^41^ ^]^ Lu et al. claimed repeatable quaternary memory behavior from MASnBr_3_‐based memcapacitors (ITO/MASnBr_3_/Au).^[^ ^42^ ^]^ Four capacitive states could be modulated from 0–169 pF through modulating the applied voltages (Figure 6b). Remarkably, almost all of the devices showed quaternary switching (yield ≈ 100%), in which each state could be sufficiently distinguished from a narrow distribution and maintained for 10^4^ s. Later, they obtained four resistive states again from the memristor with the same device structure (ITO/MASnBr_3_/Au) (Figure 6c).^[^ ^43^ ^]^ The memristor possessed a well‐separated current ratio (10^2^–10^3^) and 10^4^ endurance cycles, indicating the reliable stability of the formation/rupture of CFs caused by the migration of Br^−^ ion under an electric field. From this standpoint, more OHP materials with multilevel storage properties are expected to be explored for high‐performance RRAM.

### 5.2 Multifunctional RRAM

In addition to the basic resistive switching behaviors, the RRAM devices can implement some exotic multi‐functionalities by exposing to special stimuli.^[^ ^44^ ^]^ For example, Wu et al. proposed to use a photocatalytic hydrolysis reaction to tune the RS behavior in the presence of water molecules.^[^ ^45^ ^]^ The fabricated water‐coupled Ag/TiO_2_‐graphene‐TiO_2_/Al memristor exhibited current ratio from 5 to 44, and promoted three separate resistance, showing great potentials for humidity sensors and high‐density memory applications.^[^ ^46^ ^]^ Bae et al. developed an ionic‐liquid‐based soft memory device for fluid‐based logic operations using a Cu/(PAANa^+^:H_2_O):(NaOH)/Cu structure in a cylindrical microchannel.^[^ ^47^ ^]^ Zhou et al. found that Ag/TiO_x_ nanobelt/Ti memory device could evolve from a non‐standard faradic capacitance in dry circumstance to a battery‐like capacitance at a moisture atmosphere (35–45%), and finally to the RS state at the RH of 95–100%.^[^ ^48^ ^]^ OHPs materials, in particular, being much sensitive to oxygen, H_2_O and light, can definitely serve as promising proxies for multifunctional RRAM applications.

#### 5.2.1 Capacitive Effect

**Figure 7:**
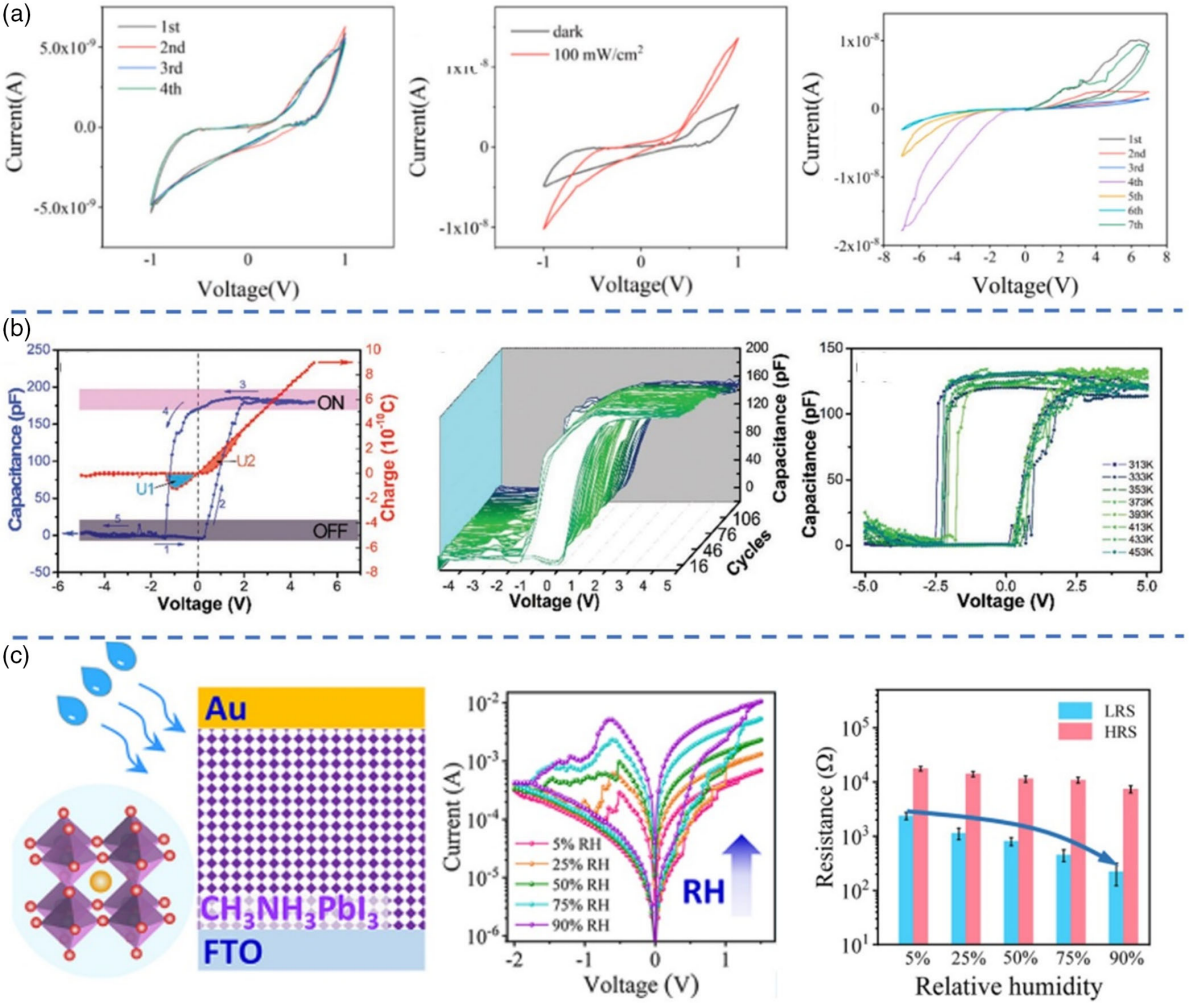
a) Typical *I–V* curves of Al/Cs_0.05_(FA_*x*_MA_1−*x*_)_0.95_PbBr_*y*_I_3−*y*_/TiO_2_/FTO device under different light conditions. Reproduced with permission.^[^ ^49^ ^]^ Copyright 2021, Elsevier. b) Memcapacitive characteristics of ITO/MASnBr_3_/Au device. Reproduced with permission.^[^ ^42^ ^]^ Copyright 2019, Wiley‐VCH. c) The schematic diagram and RS curves of Au/MAPbI_3_/FTO device under different RH condition. Reproduced with permission.^[^ ^38^ ^]^ Copyright 2021, American Chemical Society.

Apart from the conventional hysteresis phenomena, some other optoelectronic properties can also be observed in OHPs‐based optoelectronic coupling memristors, such as capacitive effect. Wang *et al.* reported a triple cation OHP material with the structure of Cs_0.05_(FA_*x*_MA_1−*x*_)_0.95_PbBr_*y*_I_3−*y*_, where the light‐ and voltage‐response presented a synergistic effect, transforming from capacitive to memristive behavior.^[^ ^49^ ^]^ As shown in **Figure** **7**a, the reliable RS behavior could be obtained with low power consumption (≈10^−9^ W at ±1 V) and high HRS/LRS ratio (≈10^3^). Compared to the dark operating condition, the hysteresis loop became smaller under the condition of illumination. The capacitive‐coupled behavior (CCB) appeared due to the formation of the built‐in electric field under a low voltage (±1 V). However, with the help of ions and vacancies‐based CFs, the CCB can be transformed to memristive behavior at a higher voltage (±7 V). Besides, at high voltage, the current tended to decrease due to the movement of fewer ions and vacancies. In this case, dual‐regulation of varing voltage and light conditions resulted in the switching of memristive and capacitive coupling behaviors. As reported previously, the similar physical dynamic processes of transition state metal oxides‐based devices can be explained by H_2_O adsorption or H_2_O redox reaction, leading to ion migration for RS memory.^[^ ^48^ ^]^

Despite several cases of capacitive behaviors accompanied by memristive switching, the independent memcapacitive switching behavior is relatively rare. Lu et al. reported a new type of independent memcapacitor devices based on lead‐free MASnBr_3_ for the first time. As shown in Figure 7b, the ITO/MASnBr_3_/Au devices exhibited apparent C–V hysteresis and Q–V loops, conforming well to the p‐n model by the equation^[^ ^42^ ^]^

(1) $$\frac{1}{C^{2}}=\frac{2\left(V_{b}-V\right)}{A^{2}e\varepsilon \varepsilon_{0}N_{0}}$$

Where V_b_, V, A, ε, ε_0_, and N_0_ represent built‐in potential, applied voltage, the device area, relative permittivity, vacuum permittivity, and doping concentration, respectively. Different from the classical p–n junction, this p‐i‐n junction capacitance performance was attributed to Br^−^ migration and oxidization around the interface of OHPs/Au electrodes. Wu et al. also proved that the perovskite bulk transport and the Schottky barrier at the MAPbBr_3_/ITO interface could be reversibly regulated, which was different from the filament‐induced switching behavior of the MAPbI_3_‐based device.^[^ ^26^ ^]^ However, for the capacitive effect in OHPs‐based devices, further experimental proofs and theoretical physical models are in urgent need. Considering that ion migration is a quite common phenomenon in OHPs, more capacitive behaviors and capacitive devices are anticipated to be discovered for future electronic applications.

#### 5.2.2 Humidity Effect

Recently, several studies have demonstrated that the humidity effect plays an obvious role for OHPs‐based materials and OHPs‐based resistive switching characteristics. On one hand, when exposed to high humidity, a number of OHPs can become unstable, which leads to degradation or counter‐electrode reactions. For example, the typical OHP of MAPbI_3_ often undergoes severe degradation under humidity conditions, where it may decompose to CH_3_NH_2_, HI, and PbI_2_. Some possible hypotheses of the degradation mechanism have been proposed over the years. It was deduced that ionic crystal perovskite materials are composed of relatively weak hydrogen‐bonding interaction between MA^+^ and PbI_6_ octahedra. When they encounter moisture, PbI_6_ octahedra may form a stronger hydrogen bonding interaction with H_2_O than MA^+^, inducing the degradation process.^[50a]^ On the other hand, some researchers have also reported that the device performance, ion migration and charge‐generating effect could be improved under appropriate humidity conditions. Recently, Zhang et al. explored the RS characteristics and working mechanism of a flexible Au/CH_3_NH_3_PbI_3_/FTO memristor at different humidity environments (5–90% RH).^[^ ^38^ ^]^ It was found that the memristor system was in a regular operation at the condition of RH < 75%, while a rapid increase of conductivity appeared at a higher RH of 90%. Additionally, as shown in Figure 7c, with the increase of the RH level, a pronounced increase in LRS current was observed, which could be ascribed to the water‐induced reduction of the iodide migration barrier. When water molecules moved into the HPs crystal lattice and were bound to MA^+^ cations, the PbI_6_ cage might be distorted and/or expanded, causing a weakened Pd‐I bond and decreased migration barrier. Thus, more iodine vacancies could be produced to induce a large‐sized conductive channel and higher LRS current. This hypothesis was well confirmed by humidity‐dependent Raman spectroscopy and XRD measurements in this work. The humidity‐sensitive RS devices based on OHPs hold great promise for sensing and security applications. Since the humidity effect is a double‐edged sword for OHPs application, more exploration concerning OHPs‐based memristors and their working mechanisms need to be further conducted.

#### 5.2.3 Light Response

**Figure 8:**
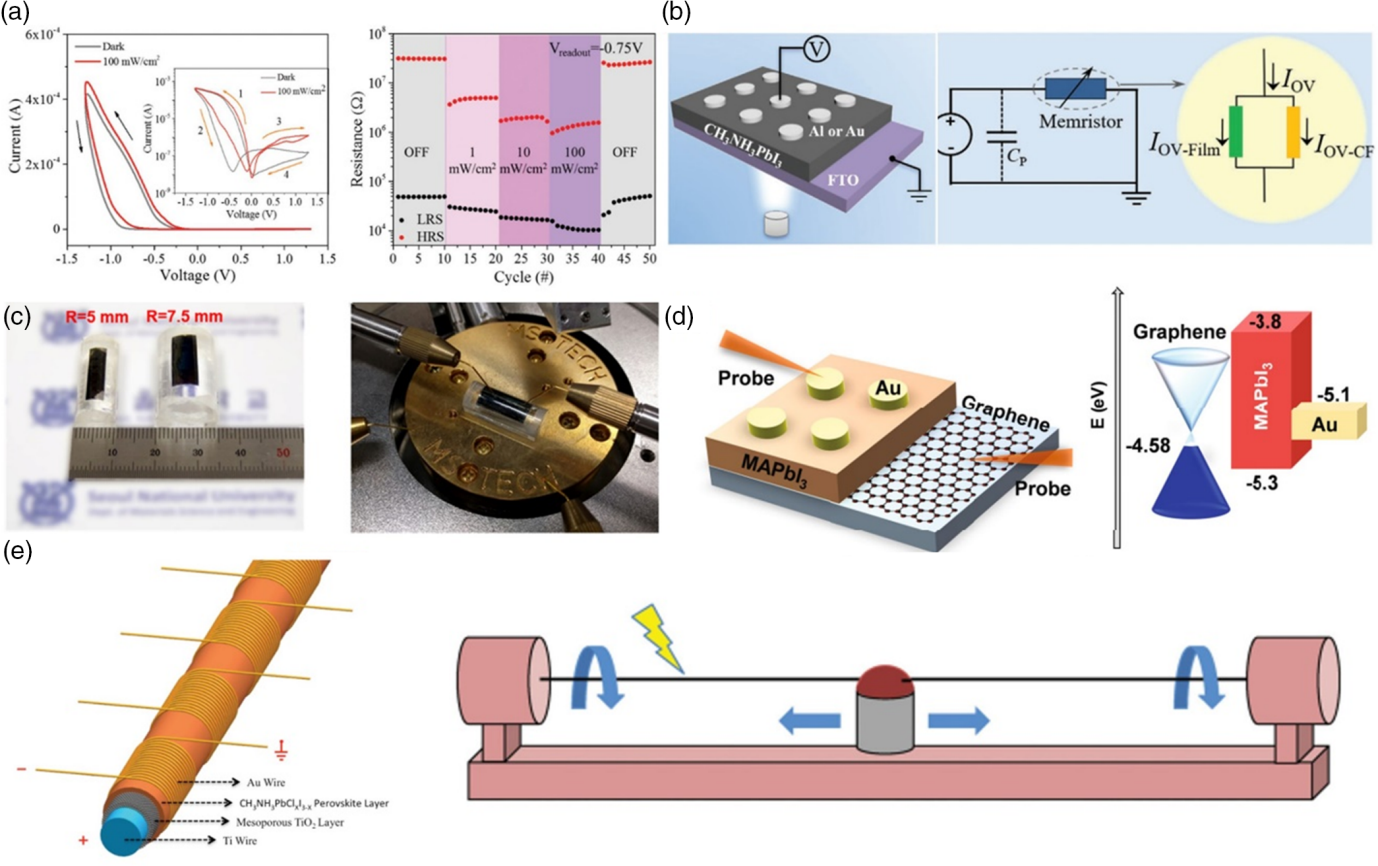
a) Switching characteristics of Au/MAPbBr_3_/ITO device in the dark and under UV light. Reproduced with permission.^[^ ^26^ ^]^ Copyright 2018, Wiley‐VCH. b) Equivalent circuit diagram of RRAM device with *I*_OV_ effect. Reproduced with permission.^[^ ^52^ ^]^ Copyright 2020, Wiley‐VCH. c) Photographs of the device at the bending condition. Reproduced with permission.^[^ ^33^ ^]^ Copyright 2017, American Chemical Society. d) Schematic illustration and energy bandgap of Au/MAPbI_3_/MLG‐TCE device. Reproduced with permission.^[^ ^36^ ^]^ Copyright 2018, Springer Nature. e) Schematic illustration of a fiber‐shaped OHP RRAM device and the homemade wire coating machine. Reproduced with permission.^[^ ^54^ ^]^ Copyright 2016, Wiley‐VCH.

Taking the photoactive nature of OHPs into consideration, light stimuli can be employed to modulate RS behavior of the OHPs‐based device.[^44^, ^51^] Generally, under an applied light illumination and a built‐in electric field, photogenerated carriers can generate in the OHPs layer and drift in the depletion region to cause a raised photocurrent, as well as an increased HRS or LRS current state. Wu et al. attempted to exploit the photoresponsive functionality of the Au/MAPbBr_3_/ITO devices. By adjusting the UV light intensity ranging from OFF or 1 to 100 mW cm^−2^, the HRS and LRS current level could be readily changed and recovered (**Figure** **8**a). The photoresponsive experiments can also be adopted as convincing evidences to exclude the probability of metallic filaments from the mechanism level.^[^ ^26^ ^]^ To our knowledge, the overshoot current (*I*_OV_) in the RRAM set process usually promotes the formation of CFs under an applied electric field, which may decrease the RS performance and result in high power consumption. Liu et al. claimed that the visible light irradiation helped to suppress the *I*_OV_ during the electroforming process.^[^ ^52^ ^]^ Figure 8b shows an equivalent circuit diagram of the RRAM system with the *I*_OV_ effect. The memristor was connected in parallel with a parasitic capacitance (*C*_P_), which can discharge from RRAM during the initial electroforming process, inducing the appearance of *I*_OV_

(2) $$I_{\text{OV}}=C_{p}\frac{dV_{\text{Forming}}}{dt}$$

(3) $$I_{\text{OV}}=I_{\text{OV}-\text{CF}}\;+\;I_{\text{OV}-\text{FILM}}$$

The d*V*_forming_/d*t* represents the voltage transient during the electroforming process. According to these equations, they tried to facilitate the ion migration in MAPbI_3_ through light irradiation, aiming to decrease *V*_Forming_ and increase *I*_OV‐Film_ (i.e., film conductivity). As a result, *I*_OV_ was reduced by restraining the CFs growth (i.e., *I*_OV‐CF_). The MAPbI_3_‐based devices achieved superior performances with an operating current of 0.06 mA and power consumption of 0.12 mW, which were much lower than those of previously reported devices. However, it should be noted that the OHPs are often faced with the risk of degradation in the presence of light in air condition, which can be ascribed to the weak interaction between organic spacer and octahedra group. When the light stimuli is utilized to fabricate multifunction RRAM devices, the experimental process should be proceeded under mild light illumination and vaccum conditions.

#### 5.2.4 Flexible RRAM

Recently, flexible electronics (i.e., foldable, stretchable, bendable, and wearable devices) have become a hot topic.^[^ ^53^ ^]^ A variety of materials have been utilized for flexible RRAM with state‐of‐art experimental demonstrations. Apart from the basic electrical properties, for flexible devices, the endurance ability and retention time play a vital role in the practical evaluation. It has been studied that the endurance property mainly depends on the film quality, thickness and morphology before and after bending. OHP materials with ultra‐flexibility and close packing have also been applied to prepare flexible RRAM devices. In 2016, a flexible MAPbI_3_‐based device with the structure of Au/MAPbI_3_/ITO/PET was prepared by Lee et al. The flexible OHPs RRAM device showed an operational voltage as low as 0.7 V and retention time over 10^4^ s even under tensile and compressive conditions. In their work, the working mechanism was explained by the migration of iodide vacancies (*V*_I_) and the formation of CFs.^[^ ^32^ ^]^ Afterward, Jang et al. focused on modulating the OHPs defects and film morphology (e.g., grain size and peak‐to‐valley depths) to optimize the flexible RS performance. By adding a suitable amount of hydrogen iodide (HI) into OHP of MAPbI_3_, a smooth OHP thin film was achieved with a small crystal grain size.^[^ ^33^ ^]^ The flexible Ag/MAPbI_3_/Pt/COP (a cyclo‐olefin polymer) afforded 1300 cycles of endurance, including 836 cycles with a bending radius of 7.5 mm and 452 cycles with a bending radius of 5 mm (Figure 8c). More importantly, the device showed excellent *I–V* performance, including ON/OFF ratio of 10^6^ and fast operational time in milliseconds.

Besides the commonly‐used FTO/PET or ITO/PET electrodes, the multilayer‐graphene (MLG) transparent conductive electrodes (TCEs) have also been applied for flexible OHPs‐based RRAM. Jang et al. prepared a MLG‐TCE/MAPbI_3_/Au sandwich device, showing a reversible bipolar RS behavior with set/reset voltage of 0.6/−0.5 V.^[^ ^36^ ^]^ Even after being bent for 1000 cycles at a radius of 4 mm and a bending frequency of 0.5 Hz, the flexible device could maintain the performance with retention time of 10^4 ^s and endurance cycles of 500 (Figure 8d). In their study, the HRS and LRS was explained by Ohmic and space‐charge‐limited conduction (SCLC) mechanisms, which could be ascribed to the *V*_I_ and the charge trapping/detrapping effect under the applied electric field. Furthermore, fiber‐shaped devices own a bright future for wearable electronics and adaptability to e‐textiles, due to their intrinsic bendable/stretchable structure. Zou et al. developed a free‐standing fiber‐shaped RRAM device, as shown in Figure 8e.^[^ ^54^ ^]^ The mesoporous TiO_2_ layer was introduced to enhance the surface tension effect of MAPbCl_*X*_I_3−*X*_ precursor solution. By dip‐coating with a balanced coating speed and a standing time for drying, multilayer perovskites were coated on the Ti/TiO_2_ electrode. The as‐fabricated OHP‐based RRAM device presented a bipolar RS feature with the Set/Reset voltage of +1.0/−1.58 V and retention time of 10^4^ s. The flexible fiber‐shaped RRAM system indicated the potential application in large‐scale manufacture and wearable e‐textile.

### 5.3 Artificial Synapses

**Figure 9:**
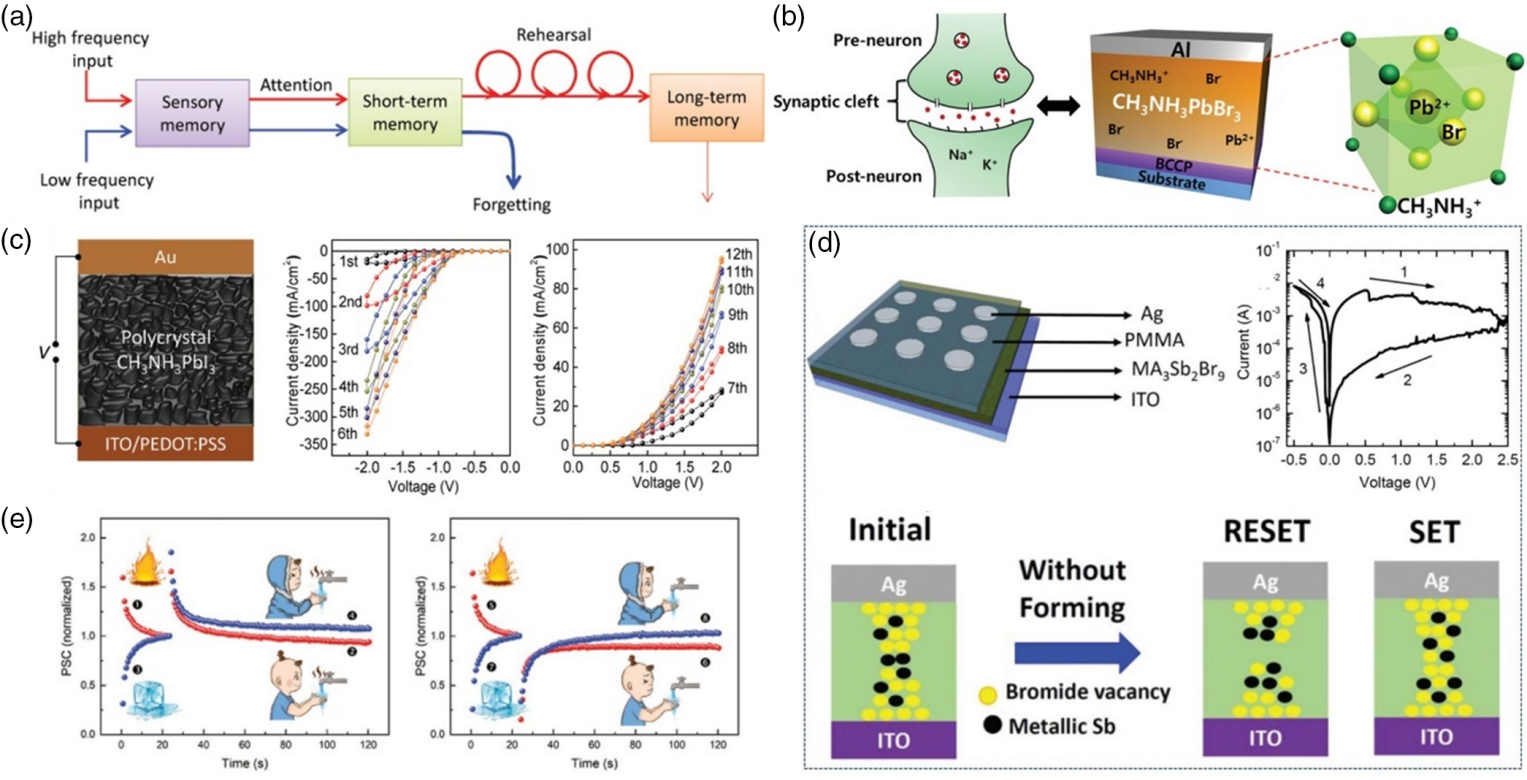
a) Schematic diagram of the psychological model of human memory. Reproduced with permission.^[^ ^15^ ^]^ Copyright 2016, Wiley‐VCH. b) Schematic illustration of a MAPbBr_3_‐based artificial synapse. Reproduced with permission.^[^ ^57^ ^]^ Copyright 2016, Wiley‐VCH. c) Schematic illustration and memristive characteristics of a MAPbI_3_‐based synaptic device. Reproduced with permission.^[^ ^15^ ^]^ Copyright 2016, Wiley‐VCH. d) Schematic illustration, *I–V* characteristics and switching mechanism of Ag/PMMA/MA_3_Sb_2_Br_9_/ITO device. Reproduced with permission.^[^ ^59^ ^]^ Copyright 2019, Royal Society of Chemistry. e) Experimental emulation for nociceptors based on metaplasticity. Reproduced with permission.^[^ ^16^ ^]^ Copyright 2020, Wiley‐VCH.

With the development of big‐data and AI era, massive data storage and processing are in urgent need. However, the traditional Von Neumann computing system is difficult to meet the increasing requirement due to the separated storage and processing configuration. Therefore, the brain‐like intelligent computing system appears to be an ideal platform to break through the Von Neumann bottleneck. A series of novel electronic devices with high plasticity have been accomplished, such as synaptic bionics, neural computation, perception and motion systems.^[^ ^55^ ^]^ Especially, artificial synapses as representative candidates have been proposed to physically mimic human brain neural networks (**Figure** **9**a).^[^ ^15^, ^56^ ^]^

Because of their unique charge carrier traps, defects, ion migration and ferroelectricity, OHPs‐based memristors usually exhibit high‐performance RS behavior and hysteresis phenomenon, enabling the promising application in artificial synapses. For the first time, Lee et al. proposed an artificial synapse based on MAPbBr_3_ to emulate the working principles of biological synapses (Figure 9b), such as STP, STDP, PPF, long‐term potentiation (LTP) and excitatory postsynaptic current (EPSC).^[^ ^57^ ^]^ A vertical device of buffer‐capped conducting polymer (BCCP) electrode/MAPbBr_3_/Al was fabricated, where the two electrodes were emulated as pre‐neuron and post‐neuron, respectively, and external electrical pulses were simulated as presynaptic spikes. By tuning the pulse‐induced conductance of the OHP film, multilevel resistive states could be gained to emulate the synaptic response. Huang et al. prepared a typical MIM structured device of ITO/PEDOT:PSS/MAPbI_3_/Au. Four synaptic forms of STP, LTP, STDP and spike‐rate‐dependent plasticity (SRDP) could be mimicked by the device, displaying a much low energy consumption of femto‐Joule/(100 nm)^2^ (Figure 9c).^[^ ^15^ ^]^ Furthermore, a novel optically‐reading SRDP behavior was obtained, implying the possibility of photoelectric regulation in the device. Intriguingly, the device conductivity could be increased under both positive and negative biases to the device, which was attributed to the switchable polarity from p‐i‐n to n‐i‐p structure. Some other OHP materials, such as MAPbI_3_Cl_3‐*x*_, FAPbI_3_, and MAPbBr_3_, can also be potentially adopted for synaptic devices. Han et al. utilized a diffusive device of Au/MAPbI_3_/ITO to imitate an integrate‐and‐fire bio‐inspired neuron, which showed superior amplitude‐frequency features and highly linear conductivity modulation for more than 1000 states. Thus, the functions of the leakage, spatiotemporal integration and firing were successfully mimicked in the OHP‐based device. Additionally, a concise spiking neural network (SNN) was also constructed by integrating the ionotropic device with 2 × 2 nonvolatile Al_2_O_3_‐based synaptic array, indicating a superior selective sensitivity to a particular input.^[^ ^58^ ^]^

Park et al. reported a Pb‐free OHP material (MA_3_Sb_2_Br_9_) and fabricated MA_3_Sb_2_Br_9_‐related device of Ag/PMMA/MA_3_Sb_2_Br_9_/ITO.^[^ ^59^ ^]^ Different from the ABX_3_‐type OHPs, MA_3_Sb_2_Br_9_ showed a trigonal structure, constituted from corner‐shared SbBr_6_^3−^ octahedra and MA cations. For the RRAM application, the device showed a forming‐free bipolar RS behavior and multilevel storage characteristics with the current ratio of 10^2^, an endurance of 300 cycles, and a retention time of 10^4 ^s. Due to the presence of metallic Sb in the OHPs film, a self‐formed CF channel was formed, resulting in the forming‐free RS characteristics (Figure 9d). Moreover, artificial synaptic characteristics of LTD, LTP, STDP, EPSC, and inhibitory postsynaptic current (IPSC) were also distinctly observed. The energy consumption as low as 117.9 fJ μm^−2^ endowed the device with the potential for highly efficient neuromorphic computing. Xu et al. synthesized single‐crystalline MAPbI_3_ thin platelets and fabricated a two‐terminal lateral‐structured synaptic device with sub‐pA operating current. As shown in Figure 9e, the activity‐dependent plasticity could also be demonstrated to enable nociceptors to detect intense external harm (e.g., cold and hot stimuli).^[^ ^16^ ^]^

### 5.4 Logic Operations

To break through the limitation of Moore's law, novel logic operation concepts and integrated circuit technologies are envisioned to be proposed and developed, such as neuromorphic computing, and in‐memory computing. With the emergence and proliferating of the advanced computing paradigm, the significant issues at the same time are information security and software‐oriented security, which pushes us to exploit hardware cryptographic solutions. It is known that different cryptographic algorithms conventionally depend on one secret key, which is stored in the traditional hardware cryptographic device. In contrast, physical unclonable functions (PUFs) technology with the feature of producing random‐and‐distinct secret bit‐strings on the fly, has been regarded as one of the most promising hardware cryptographic primitives. According to the unique physical property, PUFs device can generate a secret challenge‐response pair (CRP) space, in which mathematical models (*r = f*(*c*)) can be made between inputs/challenges (*c*) and outputs/responses (*r*). Thus, for PUFs, more linearly or supra‐linearly state variations offer more ultra‐efficient information security and circuit design.

**Figure 10:**
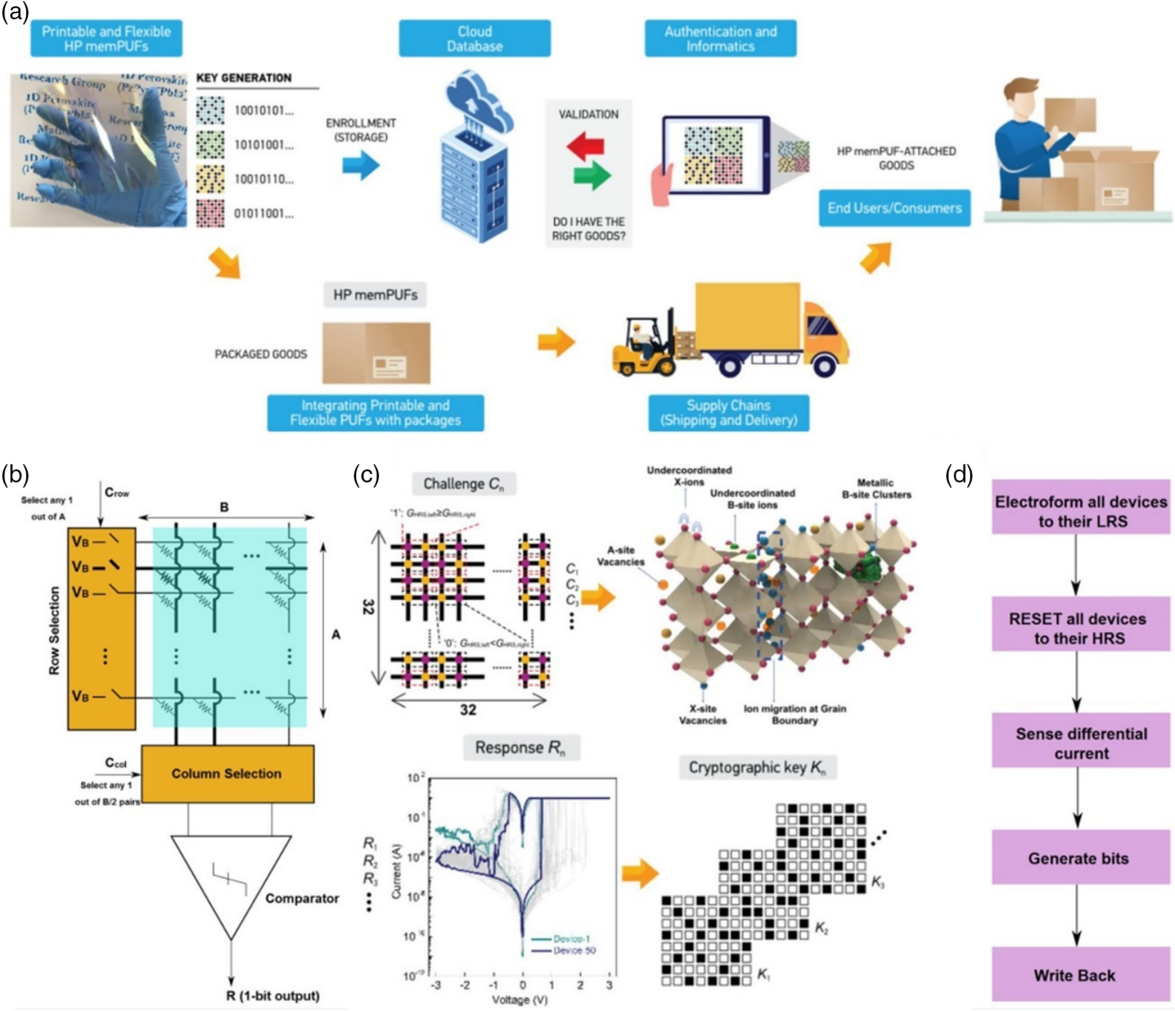
a) Schematic concept of the product authentication. b) Circuit architecture of 1D PrPyr[PbI_3_]‐based PUFs cross‐bar arrays. c) The 1D OHP cross‐bar architecture of the PUF device, and different *I–V* curves in HRS reflecting the potential of generating cryptographic keys. d) Flowchart of OHP‐based PUF bit generation. Reproduced under the terms of the CC‐BY 4.0 license.^[^ ^17^ ^]^ Copyright 2021, The Authors, published by Springer Nature.

Although silicon‐ or metallic oxide‐based PUFs have been investigated over the years, the reports concerning with organic semiconductors and OHPs remain to be rare. As discussed above, OHPs demonstrate various characteristics of facile synthesis and fabrication, modulable optical and electronic properties, etc., which can serve as sources of multidimensional entropy. Recently, Mathews et al. combined the edge‐bit‐stabilization techniques with the coupled sources of entropy in OHPs to create robust hardware security primitives, which are capable of being applied for digital fingerprints, secure key generation and device authentication (**Figure** **10**a).^[^ ^17^ ^]^ The largest implemented memristor cross‐arrays of 32 × 32 (1 kb) were constructed based on 1D pyridinium‐based OHP material (PrPyr[PbI_3_]). The architecture of the PUF device with different sensing through a low‐offset comparator was illustrated in Figure 10b. As shown in Figure 10c, the high current ratio, exalted uniformity and reliability of the primitive were obtained. Figure 10d depicted a flow chart of OHP‐based PUFs bit generation combining with a different write‐back strategy. Furthermore, the reconfiguration of the PUF key could also be achieved to enable freshness of the cryptographic key, which was confirmed by the stark difference in resistance distributions and cycle‐to‐cycle writing variability after reconfiguration. Compared to most mature RRAM PUFs or non‐CMOS reconfigurable PUFs, the 1kb OHP‐based PUF device offered a state‐of‐the‐art demonstration with excellent reconfigurability and write‐back efficiency.^[^ ^60^ ^]^

## 6 Mechanisms and Performance Improvement Strategies

### 6.1 Working Mechanisms

**Figure 11:**
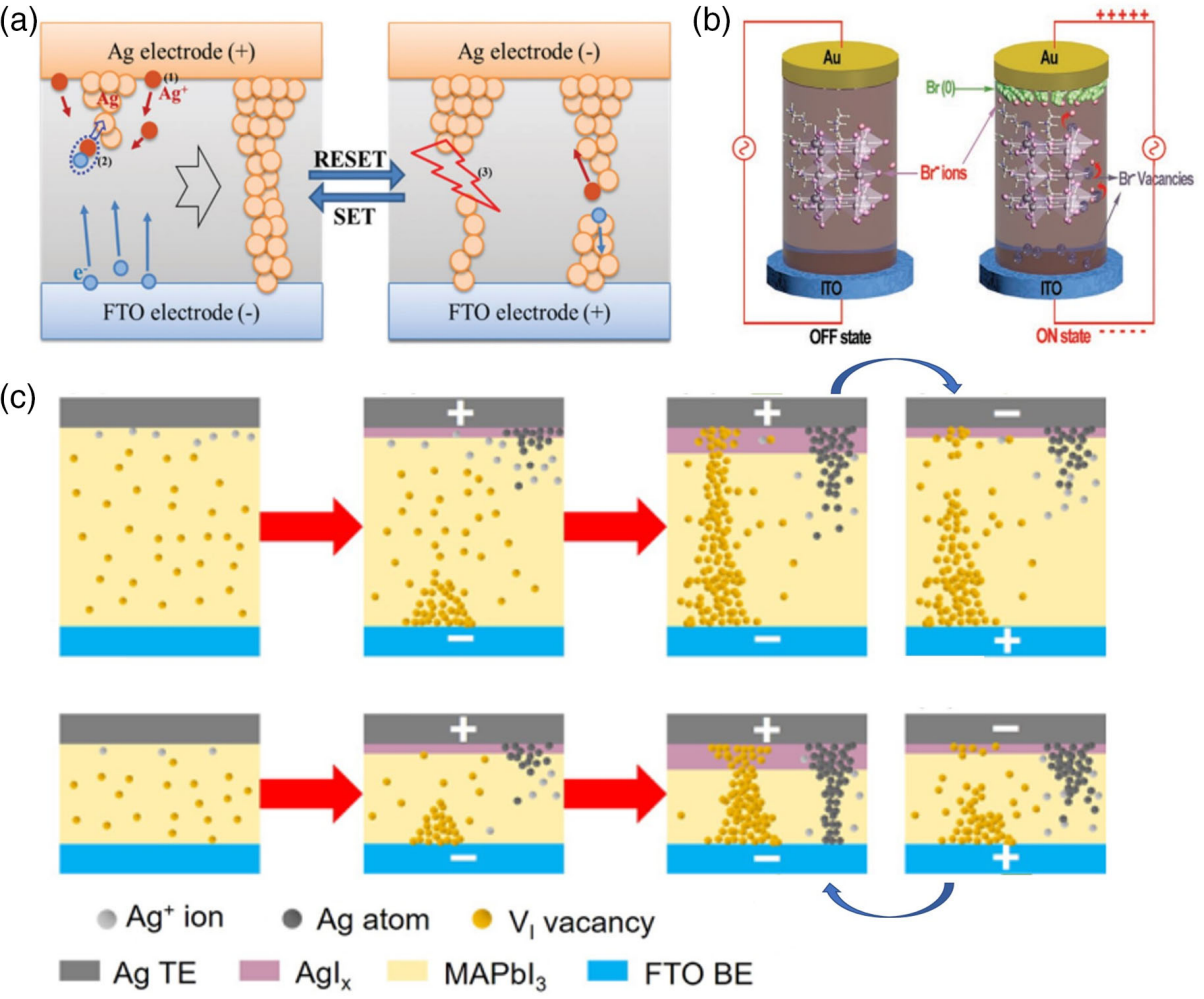
a) Schematic diagram of the electrochemical formation and rupture of Ag‐based CFs. Reproduced with permission.^[61b]^ Copyright 2016, Royal Society of Chemistry. b) Schematic diagram of Br^−^ migration. Reproduced with permission.^[^ ^42^ ^]^ Copyright 2019, Wiley‐VCH. c) Double‐filament model of the device. Reproduced with permission.^[^ ^35^ ^]^ Copyright 2018, American Chemical Society.

Up to now, various mechanisms have been proposed to explain the hysteresis and resistive phenomenon of OHPs, such as electrochemical metallization reactions (ECM), formation of metallic clusters, ions migration, ferroelectric polarization, grain boundary effects, charge trapping/de‐trapping associated with bulk and surface defects, vacancy‐driven valence change mechanisms (VCM), and thermochemical memory effect (TCM).^[^ ^61^ ^]^ Regarding these mechanisms, ion migration of active metal ions, halide ions or vacancies is a widely accepted mechanism. A lot of studies have proposed that CFs channel may be formed due to ion migration under an applied electric field. For the metallic ion migration, the active metal atoms at the electrode/perovskite interface can be partially oxidized to generate metal cations. When the metal cations go through the perovskite layer, the electrons injected from the counter electrode may reduce the ions to metal atoms, leading to the formation of CFs.^[^ ^13^ ^]^ **Figure** **11**a illustrates the formation and rupture of the Ag filaments during electrochemical processes. When a positive bias voltage was applied, the oxidation of the Ag electrode could happen at the interface and the conducting Ag filaments could grow as Ag cations migrate from top electrode to bottom electrode. This process realized the resistance change from HRS to LRS. After applying a reverse voltage, the generated Ag filaments can be ruptured by Joule heating, causing the reduction of Ag filaments at the electrode interface and hence the transition of resistance state from LRS to HRS.^[61b]^ On the other hand, many types of defects exist in OHPs, like vacancies, cation substitutions, interstitials, and anti‐site substitutions.^[^ ^62^ ^]^ For the mechanism of halide ions and vacancies migration, halide ions usually show the low activation energy of the vacancy formation. For example, in the Au/MASnBr_3_/ITO device, Lu et al. proved that Br^−^ ion migration dominates the resistance and capacitance characteristics. As shown in Figure 11b, when the device was set to ON state, Br^−^ migration causes interstitial Br^−^ or vacancies in the film, following by accumulating the oxidation states of Br (0) to the Au electrode. Thus, the collective Br^−^ ions and vacancies generated p‐doping and n‐doping in the OHPs film, respectively, realizing a p‐i‐n junction for rapidly trapping and releasing electrons.^[^ ^42^ ^]^ Accordingly, the conduction path could be formed/ruptured under different voltage biases. In some cases, a more complicated situation may be encountered, which could not be simply classified into a specific mechanism. For instance, Pan et al. clarified a reasonable double‐filament model, for which the ion migration and metal CFs coexist in a single memory cell with Ag/MAPbI_3_/Pt structure.^[^ ^35^ ^]^ Figure 11c (top) displayed the oxidation reaction of Ag atoms into Ag^+^ and the migration of *I* ^−^ ions towards Ag electrode to form an AgI_x_ layer in a high thickness film under a positive voltage, leaving behind many vacancies that will transfer to the top Ag electrode. While in a thin film with reduced thickness (<90 nm), Ag‐based CFs dominate the conductive process (Figure 11c (bottom)). Thus, the RS behaviors of the device originate from the competition between metallic and vacancy defect CFs. Generally speaking, more experimental proofs and physical models are required for a deeper understanding of the proposed working mechanisms.

### 6.2 Performance Improvement Strategies

For practical application, more efficient and stable performance is highly desirable. So far, the traditional OHP materials are still confronted with the sensitivity to humidity, temperature, light illumination, and possible reaction with oxygen in the ambient condition. Besides, the morphology, nanostructure, film coverage and defects existing in OHP films can also play a significant role in determining the device performance. The OHP films with poor quality are likely to affect the interfacial stability and interfacial contact with the top electrode (e.g., Au, Ag, and Al). To solve these problems, the development of novel composite OHP materials, thin‐film fabrication methods, and post‐processing techniques have been spotlighted. Here, we summarize two representative strategies, i.e., componential engineering (additive technique or passivated engineering) and morphological engineering, which can lead to upgraded device performances.

#### 6.2.1 Componential Engineering

OHPs possess a unique feature of tuning their components and nanostructure by passivated engineering and additive technique. For example, Park et al. reported that the FA_0.9_Cs_0.1_PbI_3_ film exhibited a more stable performance than the pristine FAPbI_3_ film regarding the humidity‐stability test under 85% relative humidity in dark conditions. They concluded that the improved stability stemmed from the contraction of cubo‐octahedral volume.^[50b]^ Moreover, inspired by the success of the external addition of potassium halides (KI) in OHPs‐based solar cells, the doping influence of KI on the memory property has also been explored. Leu et al. prepared a ITO/PEDOT:PSS/MAPbI_3_/poly(methyl methacrylate) (PMMA)/Al device with KI as an additive.^[^ ^37^ ^]^ Compared to the pristine MAPbI_3_‐based RRAM device, KI‐doped MAPbI_3_‐based device tended to afford high‐quality films, demonstrating a superior bipolar RS performance with higher ON/OFF ratio, better retention and endurance properties. As monitored in the spin‐coating process, it was found that the PbI_2_ layer could grow into uniform crystalline grains and highly‐compact films by the addition of 5 and 7 mg mL^−1^ KI in PbI_2_. The KI‐mixed PbI_2_ film grew from pristine rod to round shape and packed tightly. The further proofs confirmed that the presence of K^+^ cation and *I* ^−^ anions helped to passivate the defects distributed at the grain boundaries and film interface. The extra *I* ^−^ anions could also compensate *I* vacancy and coordinated with Pb^2+^ in OHP films. Therefore, the conduction mechanism was ascribed to the trapping/detrapping of electrons in bulk defects, rather than the defect traps at OHP surface or interface. Besides, the other additives, such as KCl, NaCl, LiCl, and HI, can also be utilized to improve the morphology and nanostructures, as well as to suppress minor defects, which deserves further investigations. Wang et al. utilized oleic acid to passivate MAPbI_3_, leading to a superior perovskite layer with decreased defects.^[^ ^63^ ^]^ The oleic acid‐passivated device of FTO/MAPbI_3_/W exhibited excellent bipolar RS characteristics with ON/OFF ratio of 10^3^ and endurance cycles of 10^2^, which were superior to that of the pristine device.

#### 6.2.2 Morphological Engineering

To improve the films morphology, it is necessary to optimize the film fabrication techniques and post‐treatments in most cases.^[^ ^64^ ^]^ In the fabrication process, the operational parameters must be controlled in a precise way. For example, in the OSP process, the time of dripping antisolvent plays a vital role in establishing a uniform grain size and a low peak‐to‐valley depth. However, the antisolvent can also penetrate into OHP thin film at the same time if many pin‐holes exist in the film state. Besides, the annealing temperature and time can also exert an obvious effect on the OHP films. To improve film uniformity and crystalline grain size, Liu et al. applied SVA post‐treatment technique to fabricate high‐quality OHP film with large coverage and enhanced aggregation.^[^ ^23^ ^]^ During annealing progress, a mild partial pressure of DMSO vapor was introduced to modulate the crystal growth rate. Compared to the pristine film, the SVA‐treated film showed a homogeneous size and shape of crystal grains, which significantly improved the device performance.

## 7 Conclusions and Perspectives

OHP‐based materials have been recognized as rising stars to keep up with the new‐generation information technological revolution. In summary, we reviewed the recent progress of OHPs in the application of RRAM, artificial synapse, and logic operation. A concise development schedule of the OHPs‐based electronic memory devices was presented. The structural and photoelectric properties of 3D and 2D OHPs, lateral/vertical device architectures and commonly‐used device fabrication technologies were introduced. The most representative memory applications of OHPs in recent 5 years, including RRAM (binary‐, multilevel‐, and multifunctional‐RRAM), artificial synapses and neuromorphic computing, and logic operation, were summarized. In the multifunctional RRAM section, the capacitance effect, humidity effect, light‐response effect and flexibility for OHPs‐based devices were discussed. Lastly, the working mechanism exploration and performance improvement strategy were given in a recapitulative way.

Based on the research advances, it can be noted that OHPs possess distinct architectural features and physical properties of solution‐processability, tunable bandgap, excellent flexibility, abundant ions/defects, and so on. Various OHPs have been employed for RRAM, artificial synapse, and logic operation. 3D OHPs (e.g., MAPbI_3_) with the formula of ABX_3_ have attained wide attention because of their excellent optoelectronic characteristics. Besides, 3D OHPs show superior features of readily modulating the RS behaviors by external moisture or light, which can realize high‐density information storage or multifunction electronic devices. However, the poor stability limits their functionality available to more applications. By contrast, 2D OHPs possess improved stability but a reduced conductivity. Thus, seeking a balance between 3D and 2D OHPs through regulating n layers has been considered as a significant strategy for achieving optimal performance. In this review, componential engineering (i.e., additive technique or passivated engineering) and morphological engineering are concluded as two representative strategies to stabilize the OHPs and upgrade their device performance. For the operational mechanisms of OHPs‐based devices, metallic ion migration, vacancy defects, and their competition model are widely accepted. So far, OHPs‐based RRAM devices have revealed a breakthrough comparable to traditional oxides‐based performance, enabling a fast‐switching response (<100 ns), high‐density data storage (>0.1 TB in^−2^), low energy consumption (<100 fJ μm^−2^), low operational voltages (<1 V), sufficient endurance cycles (>10^5^ times), and long retention time (>10^4^ s). Although there still remains a certain distance from the silicon‐based memory devices, the OHPs‐based artificial synapses and neuromorphic computing systems have been developed to conquer the bottleneck of Von Neumann architecture. The brain‐like intelligent computing system is expected to proceed as synaptic bionics, neural computation, perception, and motion in an integrated device, which has been regarded as promising next‐generation integrated circuits of sensing, storage and computing for the upcoming big‐data and AI era.

However, for OHPs, there still exist some major limitations and challenges to be solved: a) Despite the enhanced flexibility of OHPs in the presence of organic cations, the humidity and thermal instability may cause severe damage to the device performance. Specifically, as summarized in Table 1, the endurance cycles of some OHPs‐based devices are below 1000, far from the practical applications. b) The OHPs‐based devices are normally fabricated at the order of millimeters through rough and lab‐scale processing techniques, which lag far behind large‐scale industrial production. c) Most of the research efforts focus on the requirements of theoretical study, while a set of certified standards is required to offer a fair assessment of these fabricated devices. d) More works need to be done to enable the lab‐produced device to have compatibility with CMOS or some other integrated circuits. In other words, more application scenarios of OHPs‐based RRAM devices are awaited to be created. e) The OHPs‐based devices, especially lead‐containing OHPs‐based devices still face serious issues of high toxicity to the environment, which requires to be addressed.

In the era of AI and IoT, the fast operation and high‐density memory systems are believed to act as one of the most indispensable cutting‐edge technologies in the future. With a deeper understanding and better development of OHPs, we have reason to envision that more and more advanced OHP materials that satisfy the standard of practical applications will come to the front, which will show extraordinary talents in the big‐data and post‐Moore age.

## Conflict of Interest

The authors declare no conflict of interest.

## References

[1] a) B. Zhang , W. Chen , J. Zeng , F. Fan , J. Gu , X. Chen , L. Yan , G. Xie , S. Liu , Q. Yan , S. J. Baik , Z. G. Zhang , W. Chen , J. Hou , M. E. El-Khouly , Z. Zhang , G. Liu , Y. Chen , Nat. Commun. 2021, 12, 1984; 33790277 10.1038/s41467-021-22243-8 PMC8012610

[2] a) G. Wang , Q. Zhang , F. Zhu , C. Zhang , H. Li , J. Lu , J. Mater. Chem. C 2021, 9 , 6351;

[3] a) K. Wang , S. Wang , J. Liu , Y. Guo , F. Mao , H. Wu , Q. Zhang , ACS Appl. Mater. Interfaces 2021, 13, 15315; 33760598 10.1021/acsami.1c01339

[4] a) J. Feng , Y. Jiao , H. Wang , X. Zhu , Y. Sun , M. Du , Y. Cao , D. Yang , S. Liu , Energy Environ. Sci. 2021, 14, 3035;

[5] a) F. Cao , L. Li , Adv. Funct. Mater. 2020, 31, 2008275;

[6] a) X. Yu , L. Wu , D. Yang , M. Cao , X. Fan , H. Lin , Q. Zhong , Y. Xu , Q. Zhang , Angew. Chem. Int. Ed. 2020, 59, 14527; 10.1002/anie.202005120 32506624

[7] Y. K. Wang , F. Yuan , Y. Dong , J. Y. Li , A. Johnston , B. Chen , M. I. Saidaminov , C. Zhou , X. Zheng , Y. Hou , K. Bertens , H. Ebe , D. Ma , Z. Deng , S. Yuan , R. Chen , L. K. Sagar , J. Liu , J. Fan , P. Li , X. Li , Y. Gao , M. K. Fung , Z. H. Lu , O. M. Bakr , L. S. Liao , E. H. Sargent , Angew. Chem. Int. Ed. 2021, 60, 16164. 10.1002/anie.202104812 33982380

[8] C. Ma , S. Clark , Z. Liu , L. Liang , Y. Firdaus , R. Tao , A. Han , X. Liu , L. J. Li , T. D. Anthopoulos , M. C. Hersam , T. Wu , ACS Nano 2020, 14, 3969. 32119769 10.1021/acsnano.9b07888

[9] Z. Yao , W. Zhao , S. Liu , J. Mater. Chem. A 2021, 9, 11124.

[10] a) Z. Xiao , Z. Song , Y. Yan , Adv. Mater. 2019, 31, 1803792; 10.1002/adma.201803792 30680809

[11] a) J. Choi , J. S. Han , K. Hong , S. Y. Kim , H. W. Jang , Adv. Mater. 2018, 30, 1704002; 10.1002/adma.201704002 29847692

[12] A. Younis , C. H. Lin , X. Guan , S. Shahrokhi , C. Y. Huang , Y. Wang , T. He , S. Singh , L. Hu , J. R. D. Retamal , J. H. He , T. Wu , Adv. Mater. 2021, 33, 2005000. 10.1002/adma.202005000 33938612

[13] E. J. Yoo , M. Lyu , J. H. Yun , C. J. Kang , Y. J. Choi , L. Wang , Adv. Mater. 2015, 27, 6170. 26331363 10.1002/adma.201502889

[14] J. Choi , S. Park , J. Lee , K. Hong , D. H. Kim , C. W. Moon , G. D. Park , J. Suh , J. Hwang , S. Y. Kim , H. S. Jung , N. G. Park , S. Han , K. T. Nam , H. W. Jang , Adv. Mater. 2016, 28, 6562. 27192161 10.1002/adma.201600859

[15] Z. Xiao , J. Huang , Adv. Electron. Mater. 2016, 2, 1600100.

[16] J. Gong , H. Yu , X. Zhou , H. Wei , M. Ma , H. Han , S. Zhang , Y. Ni , Y. Li , W. Xu , Adv. Funct. Mater. 2020, 30, 2005413.

[17] R. A. John , N. Shah , S. K. Vishwanath , S. E. Ng , B. Febriansyah , M. Jagadeeswararao , C. H. Chang , A. Basu , N. Mathews , Nat. Commun. 2021, 12, 3681. 34140514 10.1038/s41467-021-24057-0 PMC8211866

[18] D. Lee , B. Hwang , J. S. Lee , ACS Appl. Mater. Interfaces 2019, 11, 20225. 31117475 10.1021/acsami.9b05038

[19] a) Y. Huang , L. Tang , C. Wang , H. Fan , Z. Zhao , H. Wu , M. Xu , R. Shen , Y. Yang , J. Bian , ACS Appl. Electron. Mater. 2020, 2, 3695;

[20] a) C. Zhang , Y. Li , Q. Zhang , H. Li , Q. Xu , J. He , J. Lu , Cryst. Growth Des. 2018, 18, 1432;

[21] K. Kang , H. Ahn , Y. Song , W. Lee , J. Kim , Y. Kim , D. Yoo , T. Lee , Adv. Mater. 2019, 31, 1804841. 10.1002/adma.201804841 30932266

[22] C. Zhang , Y. Li , H. Li , Q. Zhang , J. Lu , J. Mater. Chem. C 2021, 9, 374.

[23] J. Liu , M. Ozaki , S. Yakumaru , T. Handa , R. Nishikubo , Y. Kanemitsu , A. Saeki , Y. Murata , R. Murdey , A. Wakamiya , Angew. Chem. Int. Ed. 2018, 57, 13221. 10.1002/anie.201808385 30110137

[24] F. Li , C. Zhang , J. H. Huang , H. Fan , H. Wang , P. Wang , C. Zhan , C. M. Liu , X. Li , L. M. Yang , Y. Song , K. J. Jiang , Angew. Chem. Int. Ed. 2019, 58, 6688. 10.1002/anie.201902418 30884017

[25] B. Hwang , J. S. Lee , Adv. Mater. 2017, 29, 1701048. 10.1002/adma.201701048 28558134

[26] X. Guan , W. Hu , M. A. Haque , N. Wei , Z. Liu , A. Chen , T. Wu , Adv. Funct. Mater. 2018, 28, 1704665.

[27] a) Y. Li , C. Zhang , Z. Li , P. Gu , Z. Wang , H. Li , J. Lu , Q. Zhang , J. Mater. Chem. C 2019, 7, 3512;

[28] a) Y. Li , Z. Wang , C. Zhang , P. Gu , W. Chen , H. Li , J. Lu , Q. Zhang , ACS Appl. Mater. Interfaces 2018, 10, 15971; 29682969 10.1021/acsami.8b05178

[29] a) J. Di , J. Du , Z. Lin , S. Liu , J. Ouyang , J. Chang , InfoMat 2020, 3, 293;

[30] Z. Xiao , Y. Yuan , Y. Shao , Q. Wang , Q. Dong , C. Bi , P. Sharma , A. Gruverman , J. Huang , Nat. Mater. 2015, 14, 193. 25485985 10.1038/nmat4150

[31] G. Lin , Y. Lin , R. Cui , H. Huang , X. Guo , C. Li , J. Dong , X. Guo , B. Sun , J. Mater. Chem. C 2015, 3, 10793.

[32] C. Gu , J. S. Lee , ACS Nano 2016, 10, 5413. 27093096 10.1021/acsnano.6b01643

[33] J. Choi , Q. V. Le , K. Hong , C. W. Moon , J. S. Han , K. C. Kwon , P.-R. Cha , Y. Kwon , S. Y. Kim , H. W. Jang , ACS Appl. Mater. Interfaces 2017, 9, 30764. 28825292 10.1021/acsami.7b08197

[34] J. H. Heo , D. H. Shin , S. H. Moon , M. H. Lee , D. H. Kim , S. H. Oh , W. Jo , S. H. Im , Sci. Rep. 2017, 7, 16586. 29185484 10.1038/s41598-017-16805-4 PMC5707385

[35] Y. Sun , M. Tai , C. Song , Z. Wang , J. Yin , F. Li , H. Wu , F. Zeng , H. Lin , F. Pan , The J. Phys. Chem. C 2018, 122, 6431.

[36] C. W. Jang , S. W. Hwang , S. H. Shin , S.-H. Choi , J. Korean Phys. Soc. 2018, 73, 934.

[37] C.-F. Shih , H.-T. Wu , W.-L. Tsai , C.-C. Leu , J. Alloy. Compd. 2019, 783, 478.

[38] X. Zhang , X. Zhao , X. Shan , Q. Tian , Z. Wang , Y. Lin , H. Xu , Y. Liu , ACS Appl. Mater. Interfaces 2021, 13, 28555. 34101436 10.1021/acsami.1c05590

[39] M. Saliba , T. Matsui , J. Y. Seo , K. Domanski , J. P. Correa-Baena , M. K. Nazeeruddin , S. M. Zakeeruddin , W. Tress , A. Abate , A. Hagfeldt , M. Gratzel , Energy Environ. Sci. 2016, 9, 1989. 27478500 10.1039/c5ee03874j PMC4936376

[40] a) Y. Li , Q. Qian , X. Zhu , Y. Li , M. Zhang , J. Li , C. Ma , H. Li , J. Lu , Q. Zhang , InfoMat 2020, 2, 995;

[41] B. Hwang , J. S. Lee , Nanoscale 2018, 10, 8578. 29694471 10.1039/c8nr00863a

[42] W. H. Qian , X. F. Cheng , Y. Y. Zhao , J. Zhou , J. H. He , H. Li , Q. F. Xu , N. J. Li , D. Y. Chen , J. M. Lu , Adv. Mater. 2019, 31, 1806424. 10.1002/adma.201806424 31379043

[43] W. H. Qian , X. F. Cheng , J. Zhou , J. H. He , H. Li , Q. F. Xu , N. J. Li , D. Y. Chen , Z. G. Yao , J. M. Lu , InfoMat 2019, 2, 743.

[44] a) J. Hao , Y.-H. Kim , S. N. H. S. P. Harvey , E. M. Miller , S. M. Foradori , M. S. Arnold , Z. Song , Y. Yan , J. M. Luther , J. L. Blackburn , Sci. Adv. 2021, 7, eabf1959; 33910894 10.1126/sciadv.abf1959 PMC8081365

[45] T. Guo , B. Sun , S. Ranjan , C. Du , B. Joseph Kieffer , J. Philip Mills , Y. Tong , L. Wei , Y. Norman Zhou , Y. A. Wu , Phys. Status Solidi (a) 2021, 218, 2000655.

[46] a) G. Hassan , J. Bae , M. U. Khan , S. Ali , Mat. Sci. Eng. B-Adv. 2019, 246, 1;

[47] M. U. Khan , G. Hassan , J. Bae , J. Mater. Chem. C 2020, 8, 13368.

[48] G. Zhou , Z. Ren , B. Sun , J. Wu , Z. Zou , S. Zheng , L. Wang , S. Duan , Q. Song , Nano Energy 2020, 68, 104386.

[49] S. Wang , Y. Xiong , X. Dong , J. Sha , Y. Wu , W. Li , Y. Wang , J. Alloy. Compd. 2021, 874, 159884.

[50] a) J. A. Christians , P. A. Miranda Herrera , P. V. Kamat , J. Am. Chem. Soc. 2015, 137, 1530; 25590693 10.1021/ja511132a

[51] S. Lan , J. Zhong , E. Li , Y. Yan , X. Wu , Q. Chen , W. Lin , H. Chen , T. Guo , ACS Appl. Mater. Interfaces 2020, 12, 31716. 32551530 10.1021/acsami.0c09221

[52] X. Zhao , Z. Wang , W. Li , S. Sun , H. Xu , P. Zhou , J. Xu , Y. Lin , Y. Liu , Adv. Funct. Mater. 2020, 30, 1910151.

[53] a) F. Sun , Q. Lu , S. Feng , T. Zhang , ACS Nano 2021, 15, 3875; 33507725 10.1021/acsnano.0c10049

[54] K. Yan , B. Chen , H. Hu , S. Chen , B. Dong , X. Gao , X. Xiao , J. Zhou , D. Zou , Adv. Electron. Mater. 2016, 2, 1600160.

[55] a) K. Lu , X. Li , Q. Sun , X. Pang , J. Chen , T. Minari , X. Liu , Y. Song , Mater. Horiz. 2021, 8, 447; 34821264 10.1039/d0mh01520b

[56] a) Q. Lu , F. Sun , L. Liu , L. Li , Y. Wang , M. Hao , Z. Wang , S. Wang , T. Zhang , Microsyst. Nanoeng. 2020, 6, 84; 34567694 10.1038/s41378-020-00189-z PMC8433456

[57] W. Xu , H. Cho , Y. H. Kim , Y. T. Kim , C. Wolf , C. G. Park , T. W. Lee , Adv. Mater. 2016, 28, 5916. 27167384 10.1002/adma.201506363

[58] J.-Q. Yang , R. Wang , Z.-P. Wang , Q.-Y. Ma , J.-Y. Mao , Y. Ren , X. Yang , Y. Zhou , S.-T. Han , Nano Energy 2020, 74, 104828.

[59] J. M. Yang , E. S. Choi , S. Y. Kim , J. H. Kim , J. H. Park , N. G. Park , Nanoscale 2019, 11, 6453. 30892306 10.1039/c8nr09918a

[60] M. Lee , Nat. Electron. 2019, 2, 92.

[61] a) Y. Yang , W. Lu , Nanoscale 2013, 5, 10076; 24057010 10.1039/c3nr03472k

[62] D. J. Kim , Y. J. Tak , W.-G. Kim , J. K. Kim , J. H. Kim , H. J. Kim , Adv. Mater. Interfaces 2017, 4, 601035.

[63] H. Cai , G. Ma , Y. He , C. Liu , H. Wang , Org. Electron. 2018, 58, 301.

[64] C. Zhang , H. Li , Z. Li , Y. Li , Q. J. Zhang , J. M. Lu , Macromol. Chem. Phys. 2019, 220, 1900334.

